# Type-I Interferon Signaling Is Essential for Robust Metronomic Chemo-Immunogenic Tumor Regression in Murine Breast Cancer

**DOI:** 10.1158/2767-9764.CRC-21-0148

**Published:** 2022-04-22

**Authors:** Cameron Vergato, Kshama A. Doshi, Darren Roblyer, David J. Waxman

**Affiliations:** 1 Department of Biology, Boston University, Boston, Massachusetts.; 2 Department of Biomedical Engineering, Boston University, Boston, Massachusetts.

## Abstract

Many patients with breast cancer have a poor prognosis with limited therapeutic options. Here, we investigated the potential of chemo-immunogenic therapy as an avenue of treatment. We utilized two syngeneic mouse mammary tumor models, 4T1 and E0771, to examine the chemo-immunogenic potential of cyclophosphamide and the mechanistic contributions of cyclophosphamide-activated type-I IFN signaling to therapeutic activity. Chemically activated cyclophosphamide induced robust IFNα/β receptor-1–dependent signaling linked to hundreds of IFN-stimulated gene responses in both cell lines. Furthermore, in 4T1 tumors, cyclophosphamide given on a medium-dose, 6-day intermittent metronomic schedule induced strong IFN signaling but comparatively weak immune cell infiltration associated with long-term tumor growth stasis. Induction of IFN signaling was somewhat weaker in E0771 tumors but was followed by widespread downstream gene responses, robust immune cell infiltration, and extensive, prolonged tumor regression. The immune dependence of these effective antitumor responses was established by CD8 T-cell immunodepletion, which blocked cyclophosphamide-induced E0771 tumor regression and led to tumor stasis followed by regrowth. Strikingly, IFNα/β receptor-1 antibody blockade was even more effective in preventing E0771 immune cell infiltration and blocked the major tumor regression induced by cyclophosphamide treatment. Type-I IFN signaling is thus essential for the robust chemo-immunogenic response of these tumors to cyclophosphamide administered on a metronomic schedule.

**Significance:**

Many patients with breast cancer have few therapeutic options. We show that cyclophosphamide treatment induces extensive tumor regression in a syngeneic mouse model of breast cancer via a chemo-immunogenic mechanism linked to type-I IFN production. Our findings establish that IFN signaling is essential for the robust antitumor actions of cyclophosphamide and suggest that treatment resistance may stem from silencing the IFN pathway. This suggests a new avenue for improving breast cancer treatment efficacy.

## Introduction

Despite decades of progress, there are many patients with breast cancer with limited treatment options and poor prognoses (1). One such group of patients is those with triple-negative breast cancer (TNBC), which is characterized by increased tumor aggression and poor prognosis compared with other breast cancer subtypes (2). TNBC is distinguished by the lack of estrogen receptor, progesterone receptor, and human epidermal growth factor receptor 2 (ERBB2/HER2; ref. 3), which limits treatment options. TNBC initially responds to neoadjuvant chemotherapy but often recurs and metastasizes, with poor patient prognosis. Checkpoint inhibitors are often ineffective in patients with TNBC (4, 5), despite a comparatively high mutational burden and elevated levels of tumor-infiltrating lymphocytes (6). Another group of patients with few treatment options and poor outcomes are those that acquire resistance to first-line therapies (7). For these patients, new therapeutic strategies are required to improve outcomes and extend life. The discovery and preclinical development of novel therapies is thus critically important.

Immunogenic cell death is a unique cell death mechanism that can activate both innate and adaptive immune responses (8, 9) and confer long-term immunity (10). Chemotherapy-induced immunogenic cell death is characterized by damage-associated molecular pattern responses (11), including cell surface translocation of calreticulin, a prophagocytic signal (12), release of the toll-like receptor 4 ligand HMGB1 (13), extracellular release of ATP (14), and production of type-I IFNs (15). Dendritic cells attracted by the release of these molecules by dying tumor cells in the tumor microenvironment engulf the dead and dying tumor cells and undergo maturation (9). Immunostimulatory cytokines produced by mature dendritic cells, in turn, recruit NK cells, CD4 T cells, and CD8 T cells, which can contribute to tumor regression and activate tumor-specific immunity (9). Several cytotoxic drugs approved for breast cancer have the potential to induce immunogenic cell death, including doxorubicin (16), epirubicin (17), mitoxantrone (9), and cyclophosphamide (CPA; refs. 18, 19).

Type I IFNs, primarily IFNα and IFNβ, are secreted in response to viral or bacterial infection when viral gene products or bacterial cell wall components are detected by toll-like receptors or by cytosolic sensors of specific nucleic acids (20). Type I IFNs bind to the heterodimeric IFN α/β receptor (IFNAR), which in turn activates a signaling cascade leading to increased expression of many IFN-stimulated-genes (ISG). These ISGs have diverse immunomodulatory effects, including immune cell recruitment, type II IFN production, and immune cell activation (20), opening up many novel IFN-based therapeutic opportunities for cancer treatment (21). Type I IFN signaling supports tumor cell immunosurveillance (22) and impacts the efficacy of certain anticancer therapies, including antibodies against HER2, anthracyclines, checkpoint inhibitors, and lenalidomide (23).

In murine glioma models, CPA can induce immunogenic cell death when administered on a metronomic, medium-dose intermittent chemotherapy (MEDIC) schedule (24, 25), leading to elimination of GL261 gliomas implanted in syngeneic mice and activation of long-term antitumor immunity (26). Other CPA treatment schedules are much less effective at inducing immune cell recruitment in glioma models (27), a finding that was recently validated in breast cancer models (28). CPA given on a MEDIC schedule activates tumor cell–autonomous type I IFN signaling required for CPA-induced immune cell infiltration (19), suggesting cytotoxic drug-induced type I IFN production may serve as a biomarker for the immunogenic potential of cancer cells. However, it is not known whether, and to what extent, IFN-stimulated immune cell recruitment contributes to the tumor regression induced by MEDIC CPA treatment.

Here, we investigate the immunogenic potential of CPA in two tumor models: 4T1, a Balb/c mouse syngeneic mammary carcinoma model for metastatic late-stage breast cancer (29); and E0771, a medullary breast adenocarcinoma formed spontaneously in C57BL/6 mice (30) and model for spontaneous breast cancer (31). Orthotopic E0771 tumors undergo CD8 T cell–dependent tumor regression with specific antitumor immunity when treated with doxorubicin combined with IL2 (32), but immune-based tumor regression induced by chemotherapy alone, including MEDIC scheduling of CPA (24, 25), has not been reported for either model.

We assay these lines for their capacity to mount an IFN response, as indicated by robust IFN-stimulated-gene (ISG) induction following *in vitro* treatment with activated cyclophosphamide or doxorubicin, and we assess the dependence on IFNα/β receptor-1 (IFNAR1) signaling. Furthermore, we investigate the impact of CPA administered on a MEDIC metronomic schedule on tumors implanted orthotopically in syngeneic mice. Our findings reveal a striking immunogenic response to CPA associated with increased expression of hundreds of genes, including many ISGs, and resulting in the near complete regression of E0771 tumors in a manner that is absolutely dependent on the activation of type I IFN signaling–supported immune cell recruitment.

## Materials and Methods

### Tumor Cell Lines

Cell lines were authenticated by and obtained from CH3 BioSystems (E0771, catalog no. 94A001) and ATCC (4T1 cells, catalog no. CRL-2539, RRID:CVCL_0125; B16F10 cells, catalog no. CRL-6475, RRID:CVCL_0159). Cells were not tested for *Mycoplasma*. Typically, cell lines were propagated in culture for fewer than 6–8 passages before cells were discarded and a fresh, early passage cell vial was thawed and used for experimentation. Cells were cultured in RPMI1640 (4T1, E0771) or DMEM (B16F10) medium, 10% FBS, and 1% penicillin-streptomycin at 37°C under a humidified 5% CO_2_ atmosphere. Cells were stained with 0.4% Trypan blue and counted using a Countess Automated Cell Counter (Thermo Fisher Scientific). The breast cancer cells lines used in this study were characterized with respect to their hormone receptor status and as compared with prior reports, as shown in Supplementary Fig. S1. The E0771 cells used in this study were estrogen receptor-α negative, estrogen receptor-β negative, progesterone receptor negative, and Erbb2 (Her2) positive (Supplementary Fig. S1A). The same pattern of expression was observed in untreated and drug-treated E0771 cells in cell culture, and in orthotopic E0771 tumors implanted in syngeneic mice. In addition, expression patterns for progesterone receptor and for Erbb2 were similar between 4T1 and E0771 tumor cells, but differed for estrogen receptor-α and for estrogen receptor-β, which were weakly positive in 4T1 cells (Supplementary Fig. S1B), consistent with prior work (33).

### Cytotoxicity/Chemosensitivity Assay

Cells were seeded in 96-well plates, (catalog no. 10861–666, VWR) at 3,000 cells per well, 1 day prior to treatment with 10^−9^ mol/L to 10^−4^ mol/L chemically activated CPA (4-hydroperoxy-CPA, 4HC; catalog no. 19527, Cayman Chemical) or doxorubicin (catalog no. D1515 Sigma-Aldrich) for 4 hours. Cells were then washed once with PBS (catalog no. BP24384, Fisher Scientific), then cultured for 68 hours in drug-free media. Mitochondrial metabolic rate assay reagent (10 μL; catalog no. G5421, Promega) was added and then incubated at 37°C to assay cell viability. A_490_ was measured every 30 minutes (Synergy H1 plate reader; BioTek Instruments), and the timepoint where untreated cells reached A_490_ = 1.0 was used to generate dose–response viability curves and calculate IC_50_ values by nonlinear curve fitting implemented in GraphPad Prism 8 (RRID:SCR_002798).

### *In Vitro* Drug Treatment

Cells were treated for 4 hours with 4HC or doxorubicin at IC_50_ range drug concentrations using cells seeded the prior day at 50,000 (E0771) or 75,000 cells per well (4T1, B16F10) of a 6-well plate (catalog no. 10861–696, VWR). Cells were then washed once with PBS and incubated in fresh media for a total of 24, 48, and 72 hours after the start of drug treatment, at which time RNA was isolated.

### RNA Isolation and Quantitative PCR

TRIzol Reagent (1 mL; catalog no. 15596018, Invitrogen) was used to extract RNA from approximately 30–200 mg frozen tumor tissue or from cells in one well of a 6-well plate. RNA was resuspended in ultrapure water and quantified (BioTek Synergy H1 plate reader or Qubit 3.0 Fluorometer;catalog no. 15387293, Fisher Scientific). RNA (1 μg) was treated with RNase-free RQ1 DNase 1 (catalog no. M6101, Promega) with a murine RNase inhibitor (catalog no. M0314, New England Biolabs) followed by cDNA synthesis using a High-Capacity cDNA Reverse Transcription kit (catalog no. 466814, Applied Biosystems). qPCR was performed on cDNA samples using *Power* SYBR Green PCR Master Mix (catalog no. 4367659, Applied Biosystems), gene-specific primers (Supplementary Fig. S2; Eton Bioscience) and a Bio-Rad CFX384 Touch Real-Time PCR Detection System. Data for mouse ISGs (Mx1, Cxcl10, Oasl1, Cxcl11, Igtp, Rsad2), and immune marker genes (*Cd8α*, *Nkp46*, *Cd68*, *Ifng*, *Prf1*, *Gzmb*, *Cd11b*, and *Foxp3*) was analyzed by the comparative *C*_t_ method. Gene expression, normalized to 18S RNA content, was presented relative to untreated cells for *in vitro* samples, or to placebo group for *in vivo* tumor samples. Target gene–primers pairs were designed to span two adjacent exons, to be 18–22 bp long with close to 50% G:C content, and to form amplicons 50–150 bp long. Unique primer specificity was verified by extending each primer sequence by 3, 5, 10, 15, and 20 nucleotides and then using the UCSC Genome Browser BLAT tool (RRID:SCR_011919) to confirm a single correct target. Data for culture experiments are presented as mean ± SD with *n* = 2–3 replicate samples. Mouse experiments are presented as mean ± SEM for *n* tumors, as indicated. qPCR primer sequences are shown in Supplementary Fig. S2.

### *In Vitro* IFNβ Treatment

Cells seeded in 6-well plates at 200,000 cells per well were incubated overnight, then treated with recombinant mouse IFNβ1 (catalog no. 581302, BioLegend) at 28, 83, or 250 U/mL for 4 hours. Cells were then washed with PBS, fresh medium was added, and cells were harvested 2 hours later for RNA isolation.

### Poly (I:C) Transfection

Cells were transfected with 1 μg/mL poly (I:C) (catalog no. tlrl-picw, InVivogen) using 6 μg/mL poly-ethylenimine. Cells were seeded overnight in 6-well plates at 50,000 cells/well for E0771 cells and 75,000 cells/well for 4T1 and B16F10 cells. The next day, poly (I:C) (2 μL of 1 mg/mL per well) was mixed with 12 μL of 1 mg/mL poly-ethylenimine and 100 μL of serum-free media and incubated at room temperature for 15 minutes. This solution was added to 1.89 mL of full media and placed in one well of a 6-well plate for 4 hours. Cells were then washed with PBS, followed by addition of fresh media. Cells were collected 20 hours later for RNA isolation.

### *In Vitro* IFN Receptor Antibody Treatment

Cells were treated with 10 μg/mL monoclonal anti-mouse IFNα/β receptor subunit 1 (IFNAR1) antibody (clone MAR1–5A3, BioXCell), which was added to the cells together with 4HC, doxorubicin, IFNβ or poly (I:C), for 4 hours, as above. The culture medium was removed, and the cells were washed once in PBS before adding fresh media containing 10 μg/mL IFNAR1 antibody for an additional 2 to 68 hours prior to harvesting for RNA isolation.

### Conditioned Media Treatment

4T1 and E0771 cells were treated with 4HC (5 μmol/L and 4.2 μmol/L, respectively) as described above and harvested 72 hours later. Conditioned media was collected from these donor cells, transferred to drug-free (naïve) recipient cells seeded overnight in 6-well plates, and incubated for 4 hours. Cells were washed with PBS followed by replacement with fresh media prior to isolation of RNA from the recipient cells 2 hours later.

### Mouse Studies: Tumor Inoculation and CPA Treatment

Mice were treated using protocols specifically reviewed for ethics and approved by the Boston University Institutional Animal Care and Use Committee (protocol no.: PROTO201800698), and in compliance with ARRIVE 2.0 Essential 10 guidelines (34), including study design, sample size, randomization, experimental animals and procedures, and statistical methods. Six-week-old female BALB/c mice (Taconic Farms) and female C57/BL6N mice (Taconic Farms) were purchased as indicated. 4T1 cells (1 × 10^5^) or E0771 cells (2 × 10^5^) resuspended in 0.1 mL PBS were inoculated into the fourth mammary fat pad of BALB/c and C57/BL6N mice, respectively, using a 1 mL syringe (catalog no. 309628, BD Biosciences) with a 5/8-inch–long 26-gauge needle (catalog no. 305115, BD Biosciences). Tumor length and width were monitored every 3 days using a Vernier caliper, and tumor volumes were calculated: Volume = (π/6) x (*L* x *W*)^3/2^. Mice were randomized into treatment and placebo groups when average volumes reached 100–150 mm^3^ (4T1) or 200–250 mm^3^ (E0771). CPA (catalog no. C0768, Sigma-Aldrich) dissolved in sterile PBS and passed through a 0.2-μm filter was then injected intraperitoneally at 0 (vehicle control), 90, 110, or 130 mg/kg, as indicated. CPA injections were repeated every 6 days. Mice were euthanized at specified time points. Tumors were excised, washed with PBS, and flash-frozen in liquid nitrogen after placing approximately 1/3 piece of fresh tumor in 1 mL TRIzol for immediate downstream use, as required.

### Immunodepletion Studies

To deplete CD8 T cells, 0.28 mg of anti-mouse CD8α antibody (clone 53–6.7, catalog no. BE0004–1, BioXCell) or rat IgG (catalog no. I4131, RRID:AB_1163627, Sigma-Aldrich), was diluted in 0.1 mL sterile PBS then given to mice by intraperitoneal injection repeated on days -5, -1, 3, 9, and 15 (c.f., 110 mg/kg CPA treatment beginning on day 0). To achieve IFNAR1 blockade, anti-mouse IFNAR1 antibody or mouse IgG (catalog no. MS-GF-ED, Molecular Innovations) diluted in sterile PBS and injected intraperitoneally (as above) as follows: 1.0 mg on day -1, 0.5 mg on day 0, and 0.25 mg on days 3, 6, 9 and 12, with 110 mg/kg CPA treatment every 6 days, beginning on day 0.

### FACS of Tumor and Blood Samples

Approximately 1/3 of each freshly harvested tumor was dissociated to generate a 0.5 mL single-cell suspension using a Miltenyi Biotec gentleMACS Dissociator (catalog no. 130–093–235,), C-tubes (catalog no. 130–093–237), and Mouse Tumor Dissociation Kit (catalog no. 130–096–730) by using the manufacturer's instructions for “tough” tumor samples. Mouse blood obtained by tail-vein blood collection (20 μL) was placed in a 1.5 mL microcentrifuge tube with 5 μL of 1,000 U/mL heparin sodium (catalog no. H3393, Sigma Aldrich) in 0.9% saline. 1 mL of 1× RBC Lysis Buffer (catalog no. 00–4333–57, Thermo Fisher Scientific) was then added to 25 μL of each sample (dissociated tumor samples or blood samples) and shaken for 20 minutes at 20°C to destroy red blood cells. Cells were then washed with 2 mL PBS and centrifuged at 400 x *g* for 5 minutes, followed by a second wash with 3 mL Protein Extraction Buffer (0.5% BSA, 2 mmol/L EDTA in PBS, pH 7.2). The cells were spun at 400 x *g* spin for 5 minutes and resuspended in 200 μL of Protein Extraction Buffer. 100 μL was removed, mixed with 2 μL of anti-mouse CD16/CD32 antibody (catalog no. 14–0161–85, Thermo Fisher Scientific), and incubated at 4°C for 20 minutes to block nonspecific IgG binding. Anti-mouse CD8α-APC antibody (0.7 μL; catalog no. 20–1886, Clone 2.43, Tonbo Biosciences) was added and then incubated for 10 minutes at 4°C. Cells were then washed with 3 mL of Protein Extraction Buffer, spun for 5 minutes at 400 x *g*, and resuspended in 200 μL Protein Extraction Buffer. Propidium iodide (catalog no. P3566, Thermo Fisher Scientific) was added (20 ng/mL, final concentration), followed immediately by processing on a BD Biosciences FACSCalibur instrument (catalog no. 342975) and analysis using BD CellQuest Pro Software (BD Biosciences). Counted events were first gated by size based on forward scattering and side scattering parameters to omit very large and very small events. The next gate separated living from dead cells by excluding events with a propidium iodide signal. CD8α^+^ cells were then counted by excluding events lacking an APC signal. CD8α^+^ cells were presented as a percentage of total live cells.

### RNA-Seq Library Preparation and Sequence Analysis

Polyadenylated mRNA was isolated from 1 μg of total RNA from cultured tumor cells or excised tumor tissue using NEBNext Poly(A) mRNA Magnetic Isolation Module (catalog no. E7490, New England Biolabs) and the manufacturer's instructions. The resulting polyA-selected RNA was used to prepare RNA-seq libraries using the NEBNext Ultra II Directional RNA Library Prep Kit for Illumina (catalog no. E7760, New England Biolabs), NEBNext Multiplex Oligos for Illumina Dual Index Primers Set 1 (catalog no. E7600, New England Biolabs), and AMPure XP Beads (catalog no. A63881, Beckman Coulter Inc.) as per the manufacturer's instructions. Cell culture–derived RNA-seq libraries were prepared for *n* = 3 independent cultures for each condition tested, except for vehicle control–treated 4T1 cells, where *n* = 2. Tumor-derived RNA-seq libraries were prepared for *n* = 2–3 independent pools for tumor-extracted RNA for each condition tested, with each pool prepared from *n* = 2–3 independent biological replicate tumors. Libraries were multiplexed and sequenced by Novogene, Inc to an average depth of 28 million (cell culture libraries) or 13 million (tumor-derived libraries) paired-end sequence reads each (Supplementary Table S3). Data were analyzed using an in-house custom RNA-seq pipeline (35), using edgeR (RRID:SCR_012802; ref. 36) to identify differentially expressed genes for each indicated comparison, using the following cutoffs: edge R-adjusted *P* < 0.05, |fold-change| > 2.0, and fragments per kilobase per million reads (FPKM) > 0.5–1.0, as specified. Gene lists were input into the DAVID Bioinformatics Database's Functional Annotation Clustering Tool (https://david.abcc.ncifcrf.gov/home.jsp) to identify functional enrichment clusters with significance scores for each gene list. Data are presented for the top enriched term for each of the top three clusters, along with their Benjamini–Hochberg adjusted *P* values.

### Data Availability

The data generated in this study are available within the article and its Supplementary Data files. High-throughput sequencing data (Fastq files and processed data files) are available for download from Gene Expression Omnibus (RRID:SCR_005012; https://www.ncbi.nlm.nih.gov/geo/) at accession numbers GSE190531, GSE190532, GSE190533, and GSE190534.

## Results

### 4HC Induces a Type I IFN Response in Breast Cancer Lines

**FIGURE 1:**
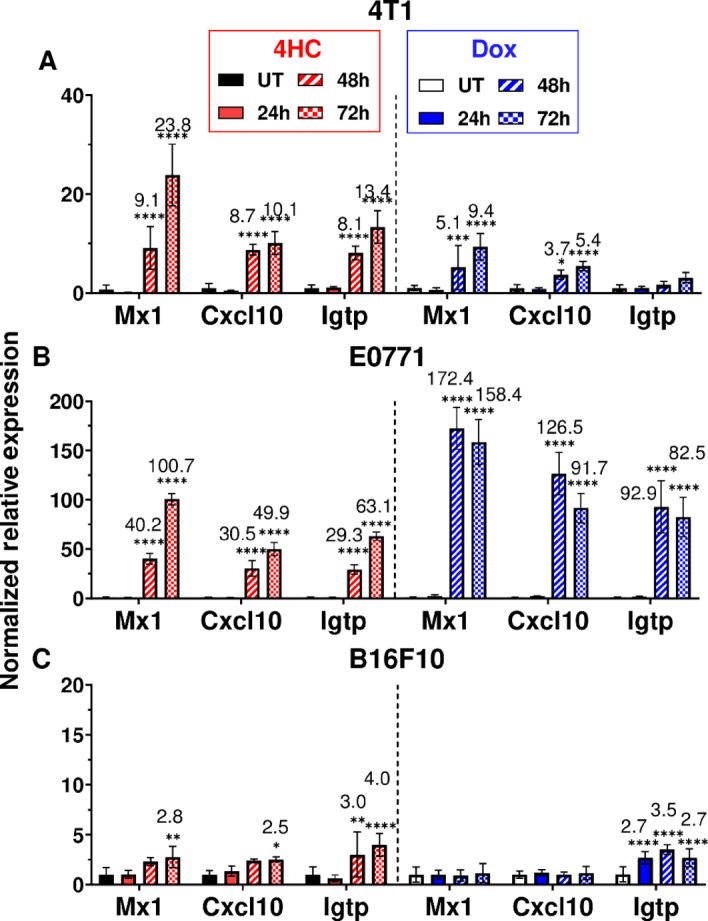
4HC and doxorubicin induction of ISGs in cultured tumor cell lines. **A,** 4T1 cells were treated for 4 hours with IC_50_ range concentrations of 4HC (5 μmol/L) or doxorubicin (2 μmol/L; see Supplementary Fig. S3), followed by removal of drug and further incubation until 24, 48, or 72 hours after initiating drug treatment. RNA was then extracted and analyzed by qPCR for expression of the three indicated ISGs. **B,** E0771 cells were treated with 4HC (4.2 μmol/L) or doxorubicin (1.7 μmol/L) as described in **A**. **C,** B16F10 cells treated with 4HC (20 μmol/L) or doxorubicin (6.6 μmol/L). Data shown are mean ± SD for *n* = 2–3 replicates, with statistical significance determined by two-way ANOVA implemented in GraphPad Prism: *, *P* < 0.05; **, *P* < 0.01; ***, *P* < 0.001; ****, *P* < 0.0001.

4T1 and E0771 breast cancer cells were treated with 4HC, an activated CPA metabolite (37), or with doxorubicin, an established immunostimulatory chemotherapeutic drug (9, 16). Drug exposures were for 4 hours (*t* = 0–4 hours) using IC_50_-range drug concentrations (Supplementary Fig. S3) to mimic *in vivo* exposure to the activated CPA metabolite 4-hydroxy-CPA, which is >90% cleared from mouse circulation within 4 hours of CPA dosing (38). Total cellular RNA was extracted and assayed for changes in the expression of several ISGs. 4HC induced strong increases in all three ISGs examined after a 24–48-hour lag, with responses being stronger in E0771 cells than 4T1 cells (Fig. 1B vs. Fig. 1A). ISG responses to doxorubicin were stronger than responses to 4HC in E0771 cells but were weaker in 4T1 cells. ISG induction was also observed when culture supernatant from 4HC-treated 4T1 and E0771 cells was applied to drug-naïve cells, indicating that the drug-treated cells secrete ISG-stimulatory cytokines (Supplementary Fig. S4), such as the type-I IFNs, IFNα and IFNβ. Weak ISG responses were seen in B16F10 melanoma cells treated with 4HC or doxorubicin at their IC_50_ concentrations (Fig. 1C; Supplementary Fig. S3C). This finding is consistent with the weak immune responses seen in CPA-treated B16F10 tumors implanted in syngeneic mice (39).

### ISGs are Induced by Multiple Signaling Pathways in 4HC-Treated Cells

**FIGURE 2:**
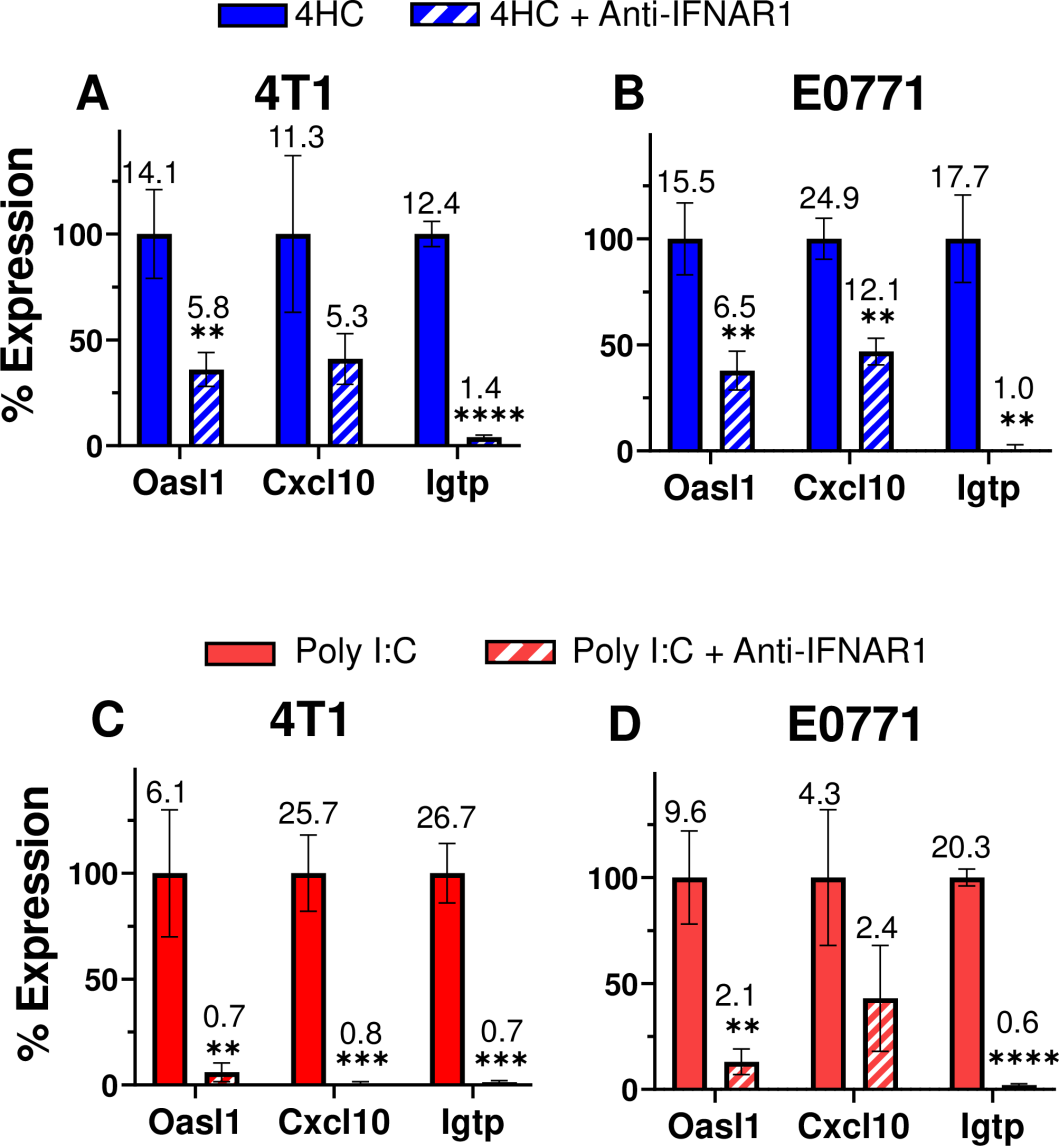
Anti-IFNAR1 antibody Inhibits ISG induction by 4HC or poly (I:C). **A** and **B,** 4T1 and E0771 cells were treated with anti-IFNAR1 antibody (10 μg/mL; Supplementary Fig. S5) in combination with 4HC using the 72-hour time-point protocol of Fig. 1, followed by qPCR analysis for ISG induction. **C** and **D,** 4T1 and E0771 cells were treated with 1 μg/mL poly (I:C) for 4 hours, alone or in combination with anti-IFNAR1 antibody (10 μg/mL), then further incubated for 20 hours in the presence of IFNAR1 antibody followed by qPCR analysis of ISG induction. Data presented are mean ± SD values for *n* = 2–3 replicates, with significance of the effect of antibody assessed by *t* test: *, *P* < 0.05; **, *P* < 0.01; ***, *P* < 0.001; ****, *P* < 0.0001. Percent gene expression was calculated as: ((x ± SD) − (z ± SD))/((y ± SD) − (z ± SD)) × 100%, where x = 4HC or poly (I:C) with anti-IFNAR1 expression value, z = untreated control expression value, and y = 4HC or poly (I:C) alone expression value. Fold change values are listed above each bar. Results shown are representative of at least two or three independent experiments.

To assess the role of type I IFN signaling in these ISG responses, 4T1 and E0771 cells were treated with 4HC in combination with anti-IFNAR1 antibody under conditions that effectively blocks direct type I IFN responses (Supplementary Fig. S5). In both breast cancer cell lines, anti-IFNAR1 antibody completely blocked 4HC induction of *Igtp*, but only partially inhibited the induction of *Oasl1* and *Cxcl10* (Fig. 2A and B). Thus, IFNAR1 signaling contributes to, but does not entirely explain, the latter two ISG responses to 4HC treatment. To further investigate the underlying mechanism for ISG induction, cells were transfected with the ds-RNA analogue, poly I:C, both with and without anti-IFNAR1 antibody. All three ISGs were induced by poly I:C in both cell lines, but only the 4T1 cell response was completely blocked by anti-IFNAR1 antibody (Fig. 2C and D). Thus, E0771 cells showed a pattern of partial inhibition of ISG induction by anti-IFNAR1 antibody with both 4HC and poly I:C. These findings are consistent with the proposal that 4HC activates a dsRNA-dependent mechanism leading to an increase in type-I IFN production and the observed downstream ISG responses. The partial inhibition by anti-IFNAR1 antibody of *Oasl1* and *Cxcl10* induction indicates these ISGs can also be formed by a type I IFN-independent mechanism in 4HC-treated cells.

### Global Effects of 4HC Exposure

**FIGURE 3:**
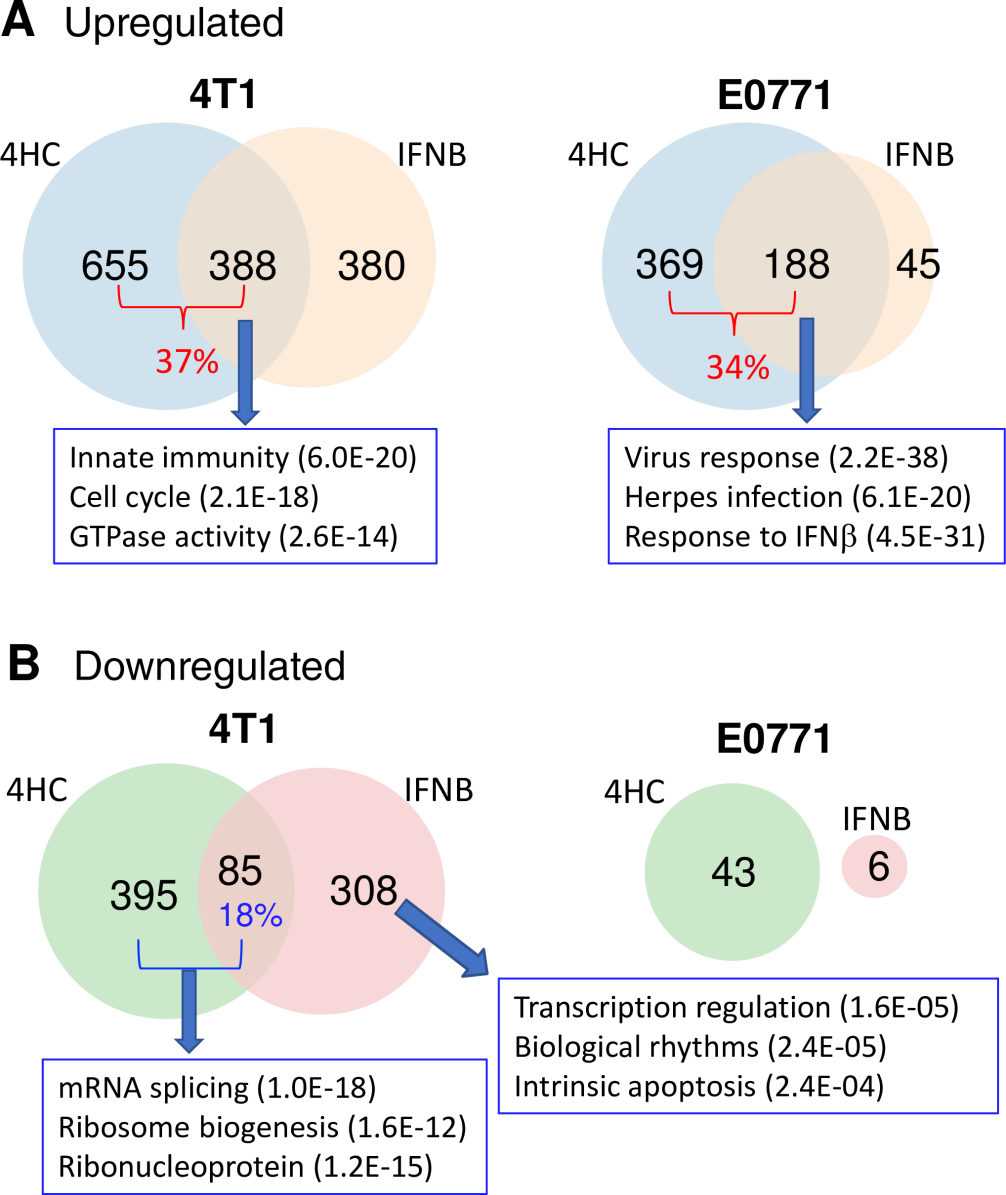
Gene responses to 4HC and IFNβ in cultured 4T1 and E0771 cells: RNA-seq analysis. Venn diagrams showing numbers of genes induced (**A**) or repressed (**B**) in cells treated with 4HC (4-hour exposure, harvest 68 hours later, as in Fig. 1) or recombinant IFNβ (4-hour exposure, 2-hour harvest 2 hours later) based on a >2-fold change in expression at FDR < 0.05. Full gene lists are shown in Supplementary Table S1. Top functional annotation clustering terms for the indicated gene sets, and their enrichment significance, are shown in boxes. The full DAVID analysis is presented in Supplementary Table S2.

RNA-seq was used to characterize the global impact of 4HC exposure on both type I IFN-dependent and type I IFN-independent genes that may potentially contribute to downstream immunostimulatory responses in each breast cancer line. In 4T1 cells, 4HC induced expression of 1,043 genes, of which 388 (37%) also responded to short-term stimulation with recombinant IFNβ, which identifies the latter genes as 4T1 breast cancer type-I ISGs (Fig. 3A). Similarly, in E0771 cells, 188 (34%) of 568 genes induced by 4HC were also induced by IFNβ (Supplementary Table S1). Top functional annotation clustering terms include innate immunity and virus response (Fig. 3A; Supplementary Table S2), consistent with 4HC inducing many type I IFN response genes in both breast cancer lines. Further supporting the proposed activation of type I IFN signaling by 4HC in both models, we identified 110 genes induced by both 4HC and IFNβ in both cell lines, and for 97 of these genes, anti-IFNAR1 antibody significantly inhibited gene induction by 4HC (Supplementary Table S1C). Finally, in 4T1 cells but not E0771 cells, many other genes were suppressed by 4HC, with enrichment for mRNA splicing and ribosome biogenesis. Notably, 85 of these genes were also suppressed by IFNβ treatment (Fig. 3B; Supplementary Tables S1 and S2).

### Metronomic CPA Induces 4T1 Tumor Growth Stasis

**FIGURE 4:**
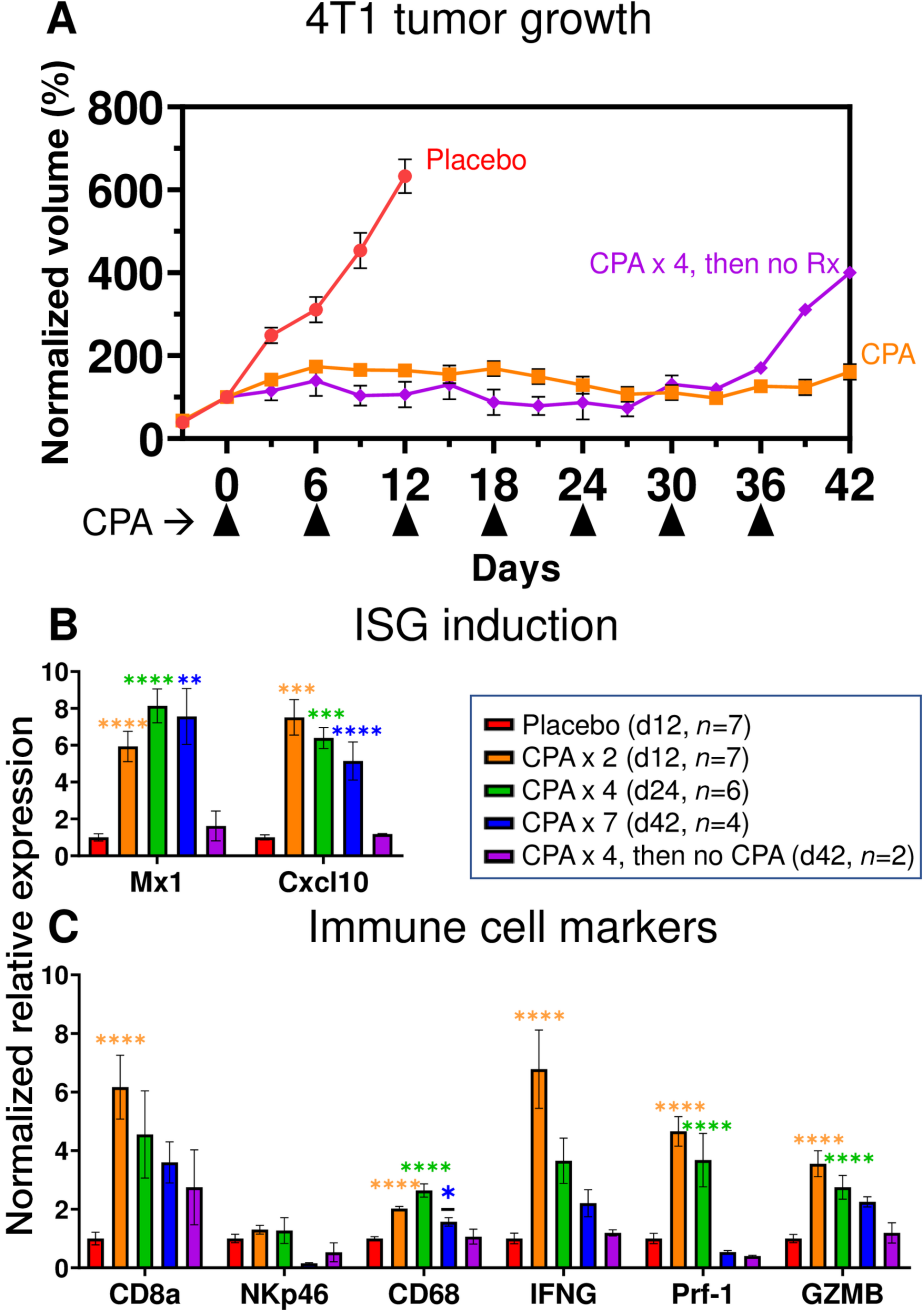
4T1 tumor growth and gene expression changes induced by CPA treatment. **A,** 4T1 cells were implanted orthotopically in 6-week-old female BALB/c mice, then treated with 130 mg/kg CPA or PBS (placebo) on a 6-day metronomic schedule once mean tumor volumes reached 100–150 mm^3^. Shown are group tumor volumes (mean ± SEM) normalized to the volume on the first day of CPA treatment (day 0). Mice were euthanized and tumors excised for qPCR analysis of tumor RNA (**B** and **C**) on treatment days 12, 24, and 42, with *n* tumors/group, as indicated (box). Thus, the curve marked CPA represents 17 tumors through day 12, then 10 tumors through day 24, and then 4 tumors through day 42 (7 CPA treatment cycles). Data indicate exponential growth of placebo group versus growth stasis through 7 CPA treatment cycles. Tumors began to regrow by day 36 when CPA was halted after four treatment cycles. **B** and **C,** qPCR analysis of ISGs and immune cell marker genes in tumor cell RNA. CPA induced tumor ISG expression after 2, 4, and 7 treatment cycles, but the induction was reversed when treatment was halted after 4 cycles. CPA induction of cytotoxic effector expression and immune cell infiltration were reduced or lost with prolonged treatment. Significance (one-way ANOVA): *, *P* < 0.05; **, *P* < 0.01; ***, *P* < 0.001; ****, *P* < 0.0001.

We investigated the impact of CPA treatment on growth of implanted 4T1 tumors, ISG expression, and immune cell recruitment. Female BALB/c mice with orthotopic 4T1 tumors were treated with CPA (130 mg/kg) or placebo (PBS) on an intermittent metronomic, 6-day repeating schedule (MEDIC schedule; ref. 24). CPA dramatically reduced the rapid growth seen in drug-free tumors (placebo group) within one treatment cycle, inducing growth stasis that persisted through 7 cycles (Fig. 4A). When treatment was halted after 4 CPA cycles, tumor growth resumed 12 days later (Fig. 4A, day 36). Analysis of total tumor RNA extracted after 2, 4, and 7 CPA cycles revealed that the ISGs *Cxcl10* and *Mx1* were initially upregulated but returned to baseline after discontinuation of CPA treatment (Fig. 4B). We evaluated tumor immune cell infiltration by monitoring changes in the expression of *Cd8a*, *Cd68,* and *Nkp46,* immune cell markers for cytotoxic T cells, macrophages, and natural killer cells, respectively. *Cd8a* showed a 6-fold increase peaking after 2 cycles then decreased with further CPA treatment, perhaps reflecting CPA-induced immune cell cytotoxicity. Similarly, *Cd68* increased 3-fold after 4 cycles then declined, while *Nkp46* showed no significant changes in expression at any time point (Fig. 4C). The immune cell effector markers *Ifng*, *Prf1* and *Gzmb* also showed peak induction after 2 CPA cycles then decreased with further treatment.

These findings were validated by RNA-seq analysis of total tumor RNA across the time course, which identified hundreds of treatment-induced gene responses. Innate immunity, immune response, and cellular response to IFNβ represent the top Functional Annotation Clusters of upregulated genes after 2, 4, and 7 CPA treatment cycles (Supplementary Table S4). Notably, these immune response gene clusters were not found in the regrowing tumors when CPA treatment was halted after 4 cycles (Supplementary Table S4). Thus, in 4T1 tumors, metronomic CPA activated a transient immune response associated with tumor growth stasis.

### Metronomic CPA Induces Robust E0771 Tumor Regression and Immune Cell Recruitment

**FIGURE 5:**
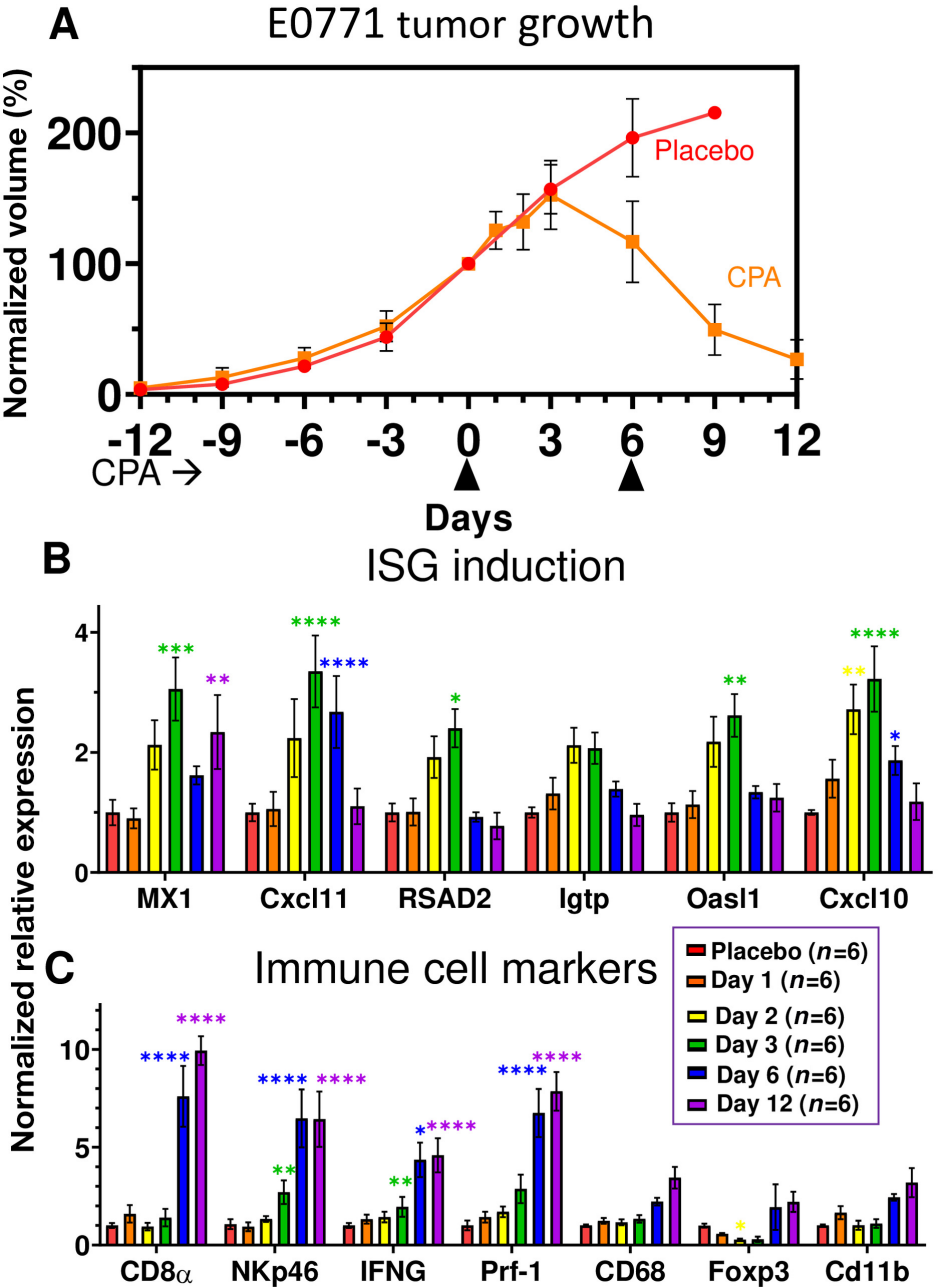
Tumor growth and gene expression changes in CPA-treated E0771 tumors. **A,** Impact of metronomic CPA treatment (110 mg/kg per injection; arrowheads along *x*-axis) on E0771 tumor growth. Data shown are mean ± SEM tumor volumes for *n* = 6 tumors per group, normalized to 100% of the day 0 volume. **B** and **C,** qPCR analysis as in Fig. 4. The weak induction of ISGs was highest three days after the first CPA dose, at which time immune cell infiltration and cytotoxic effectors were first increased and then maintained through day 12. Increases in macrophages and dendritic cells (*Cd11b*) were significant by *t* test but not by ANOVA. Significance versus placebo group (two-way ANOVA): *, *P* < 0.05; **, *P* < 0.01; ***, *P* < 0.001; ****, *P* < 0.0001.

Major regression of E0771 tumors was seen within 2 CPA treatment cycles (Fig. 5A), in contrast to the growth stasis response of 4T1 tumors. ISG induction was comparatively weak; it was first seen on day 2, peaked on day 3, then declined and was undetectable by day 12 (Fig. 5B). Importantly, ISG induction was followed by strong immune cell recruitment by days 6 and 12 (Fig. 5C). Tregs (*Foxp3*) initially decreased at day 2 before returning to basal levels by day 6 (Fig. 5C).

**FIGURE 6:**
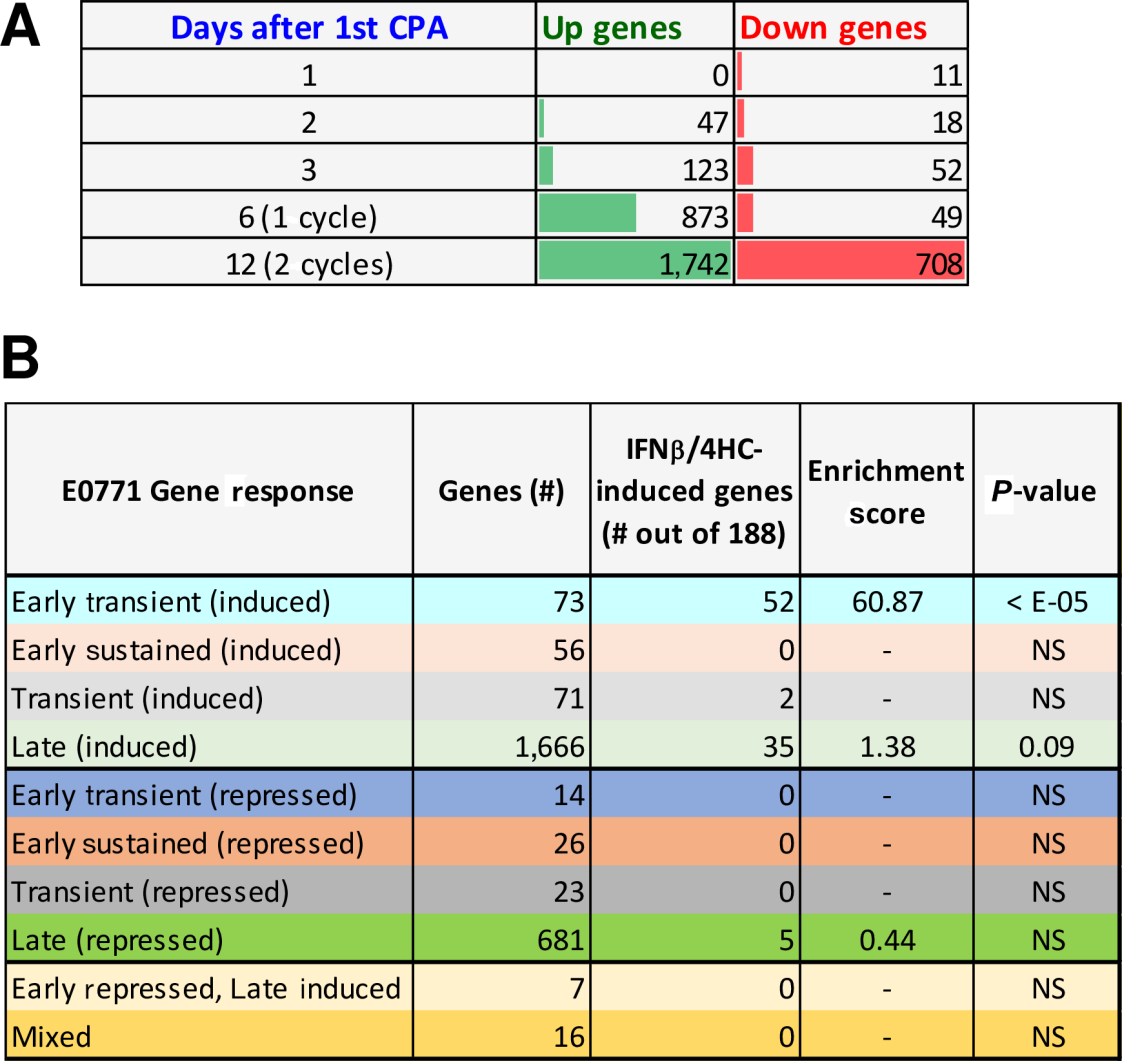
E0771 tumor RNA-seq. **A,** Number of genes showing significant upregulation or down regulation at each of five time points of metronomic CPA treatment. A total of 2,633 genes met the thresholds for a significant response (fold-change > 2 at edgeR-adjusted *P* < 0.05) at one or more time points (Supplementary Table S5A). **B,** The set of 2,633 responsive genes was classified based on the time course of response, as detailed in Supplementary Table S5B. Each set was analyzed for overlap with the set of 188 genes that were up regulated by both 4HC and IFNB in cultured E0771 cells (Fig. 3B), and enrichment scores with significance by Fisher exact text calculated compared to a background set of all genes expressed at FPKM > 1, as shown in Supplementary Table S5B.

E0771 tumor RNA-seq analysis revealed an interesting pattern: there were relatively few gene expression changes during the first 3 days after CPA treatment, followed by large numbers of treatment-responsive genes on days 6 and 12 (i.e., after 1 and 2 treatment cycles; Fig. 6A). Genes were grouped by whether their response to treatment was early (days 1–3) or late (days 6, 12), and whether the response was sustained through day 12 or was not (i.e., transient; Fig. 6B; Supplementary Table S5A and S5B). Top functional annotation clusters included innate immunity for both early-transient and late upregulated genes, whereas inflammatory response was a top cluster term for early-sustained induced genes (Supplementary Table S5C–S5F). Comparison to the set of 188 type I IFN response genes identified in cultured E0771 cells (Fig. 3A; common response to 4HC and IFNB) revealed a striking, 61-fold enrichment in the set of 73 early-transient (induced) genes, 52 (71%) of which were in the 188 gene set (*P* < E-05 vs. background set of all expressed genes; Fisher exact test). The type I IFN response genes showed no enrichment in the early-sustained induced gene set (0 of 56 genes) and marginal enrichment in the late induced gene set (35 out of 1,666 genes; 1.38-fold enrichment, *P* = 0.09; Fig. 6B). Thus, CPA induces an early type I IFN response that is not sustained through day 12, by which time there is major immune cell infiltration (Fig. 5C) and a 14-fold increase in the overall number of differentially expressed genes (Fig. 6A). Early-sustained downregulated genes were enriched for sterol and lipid metabolism, while the large set of late down genes was enriched for cell-cycle and transcriptional regulation terms (Supplementary Table S5G and S5H).

Finally, Alas2, a heme biosynthetic enzyme, and 6 hemoglobin genes (most notably four hemoglobin-beta genes) were strongly downregulated by CPA on days 1–3, but then strongly upregulated after 2 treatment cycles (Fig. 6B; Supplementary Table S5I). Hemoglobin-beta contributes to breast cancer neoangiogenesis and metastasis by a tumor cell protective antioxidant mechanism (40, 41), but also becomes a dominant self-antigen target of CD8 T cells in tumor pericytes following IL12 immunotherapy (42).

### E0771 Tumor Regression Requires CD8^±^ T Cells

**FIGURE 7:**
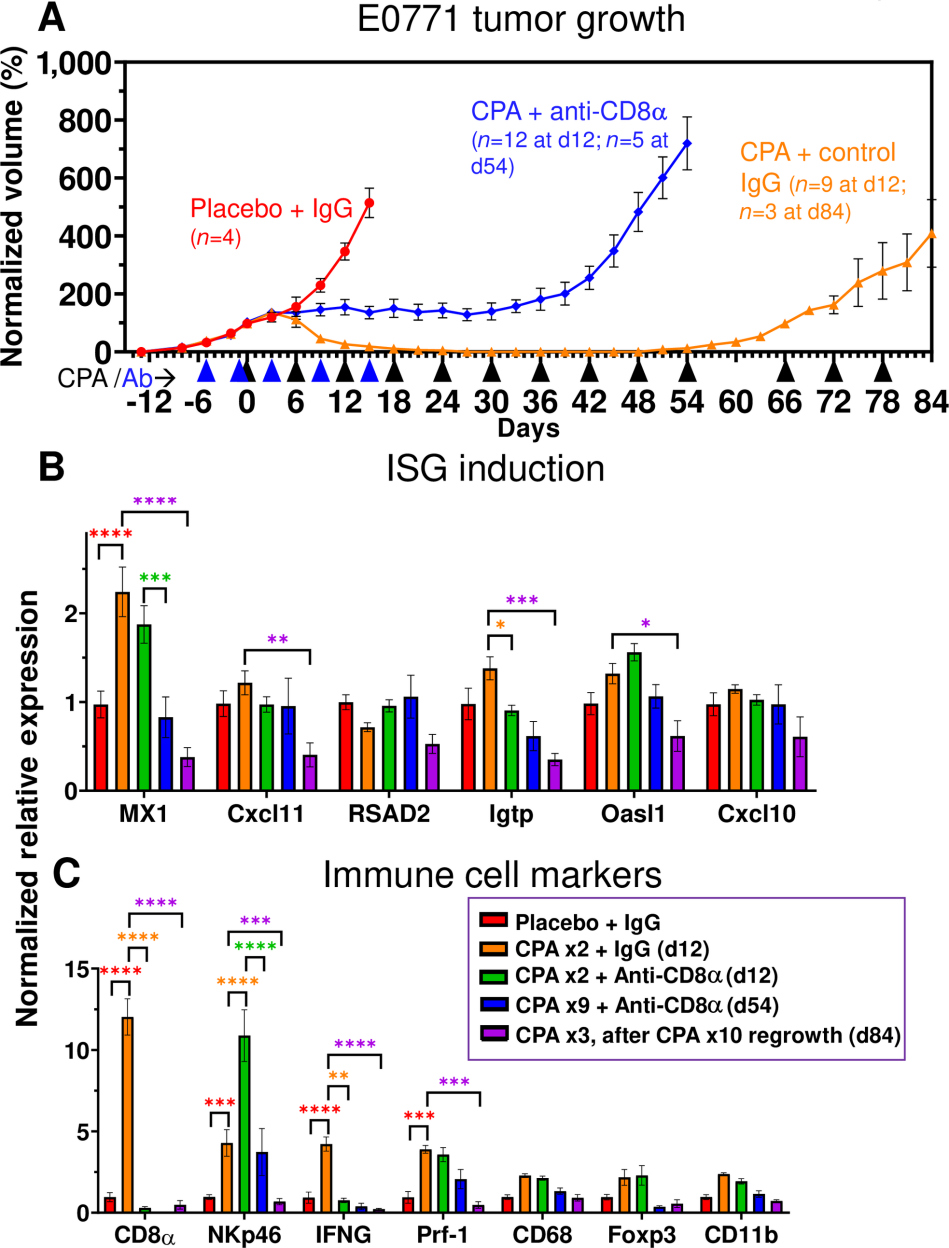
Impact of CD8α immunodepletion on CPA-induced E0771 tumor regression and immune cell recruitment. **A,** E0771 tumors were treated with CPA every 6 days at 110 mg/kg (black arrowheads along *x*-axis), alone or in combination with anti-CD8a or control IgG (blue arrowheads). Tumor volumes were normalized to the percent of day 0 volume ( = 100). By day 12, the placebo + control IgG, CPA + control IgG, and CPA + anti-CD8α groups showed three distinct growth patterns: exponential growth, tumor regression, and tumor stasis, respectively. Tumors treated with CPA + anti-CD8α resumed growth by day 42. CPA-regressed tumors eventually regrew and became resistant to CPA treatment. Data shown are mean ± SEM volumes for *n* = 4 tumors for the placebo + IgG group, *n* = 5 for CPA + IgG (d12), *n* = 3 for CPA + IgG (d84), *n* = 7 for CPA + anti-CD8α (d12) and *n* = 5 for CPA + anti-CD8α (d54). **B,** ISG induction was weak in all groups, but the regrowing CPA-resistant tumors showed decreased ISG expression. **C,** Anti-CD8α antibody prevented CPA-induced CD8 T-cell infiltration and IFNG production, but NK-cell infiltration increased. Significance (two-way ANOVA): *, *P* < 0.05; **, *P* < 0.01; ***, *P* < 0.001; ****, *P* < 0.0001.

Next, we used an immune-depletion strategy to ascertain the role of CD8 T cells in metronomic CPA-induced E0771 tumor regression. To minimize the direct effects of CPA cytotoxicity and maximize possible immune system contributions, we decreased the CPA dose from 130 mg/kg to 110 mg/kg, which was effective at inducing tumor regression, ISG induction, and immune cell recruitment (Supplementary Fig. S6). Anti-mouse CD8α antibody, or control IgG, was administered to E0771 tumor-bearing mice beginning 5 days before the first CPA treatment on day 0. FACS analysis confirmed the depletion of circulating CD8 T cells by day 1, which persisted for at least 5 weeks after the last antibody injection on day 15 (Supplementary Figs. S7 and S8). Moreover, in contrast to the near-complete tumor regression achieved in the CPA + control IgG group, an extended period of tumor growth stasis followed by robust tumor regrowth was evident in mice receiving CPA + anti-CD8α antibody (Fig. 7A). We conclude that CD8 T cells are essential for CPA-induced tumor regression, and in their absence, E0771 tumors escape the cytotoxic effects of CPA treatment.

CPA induction of the ISG *Mx1* was unaffected by anti-CD8α antibody, as was expected given the expectation that ISG gene induction occurs upstream of immune cell infiltration. Other ISGs, whose induction by CPA in E0771 tumors was transient (seen on days 2, 3, and 6, but not day 12; Fig. 5B), were not induced in the day 12 tumor samples (Fig. 7B). Importantly, anti-CD8α antibody abolished the increase in tumor-infiltrating CD8 T cells (*Cd8a*), as well as the increase in *Ifng*, which is produced by tumor-infiltrating CD8 T cells and could be an important contributor to tumor regression. Surprisingly, the induced expression of the NK-cell marker *Nkp46* was further augmented by anti-CD8α, while *Prf1*, which is produced by both CD8 T cells and NK cells, showed no net change in expression (Fig. 7C).

Regrowth of the regressed CPA + control IgG–treated tumors became apparent once CPA treatment was discontinued on day 60, after which the tumors became resistant to further CPA treatment (Fig. 7A). The expression of *Mx1* decreased below basal levels in the regrowing tumors (Fig. 7B), as did that of *Cd8a*, *Nkp46* and the cytotoxic effectors *Ifng* and *Prf1* (Fig. 7C), which may contribute to the emerging resistance to CPA. Together, these findings provide strong support for the conclusion that metronomic CPA-induced recruitment of CD8 T cells is essential for E0771 tumor regression.

### Role of Type I IFN Signaling in CPA-Induced Immune Cell Recruitment and Tumor Regression

**FIGURE 8:**
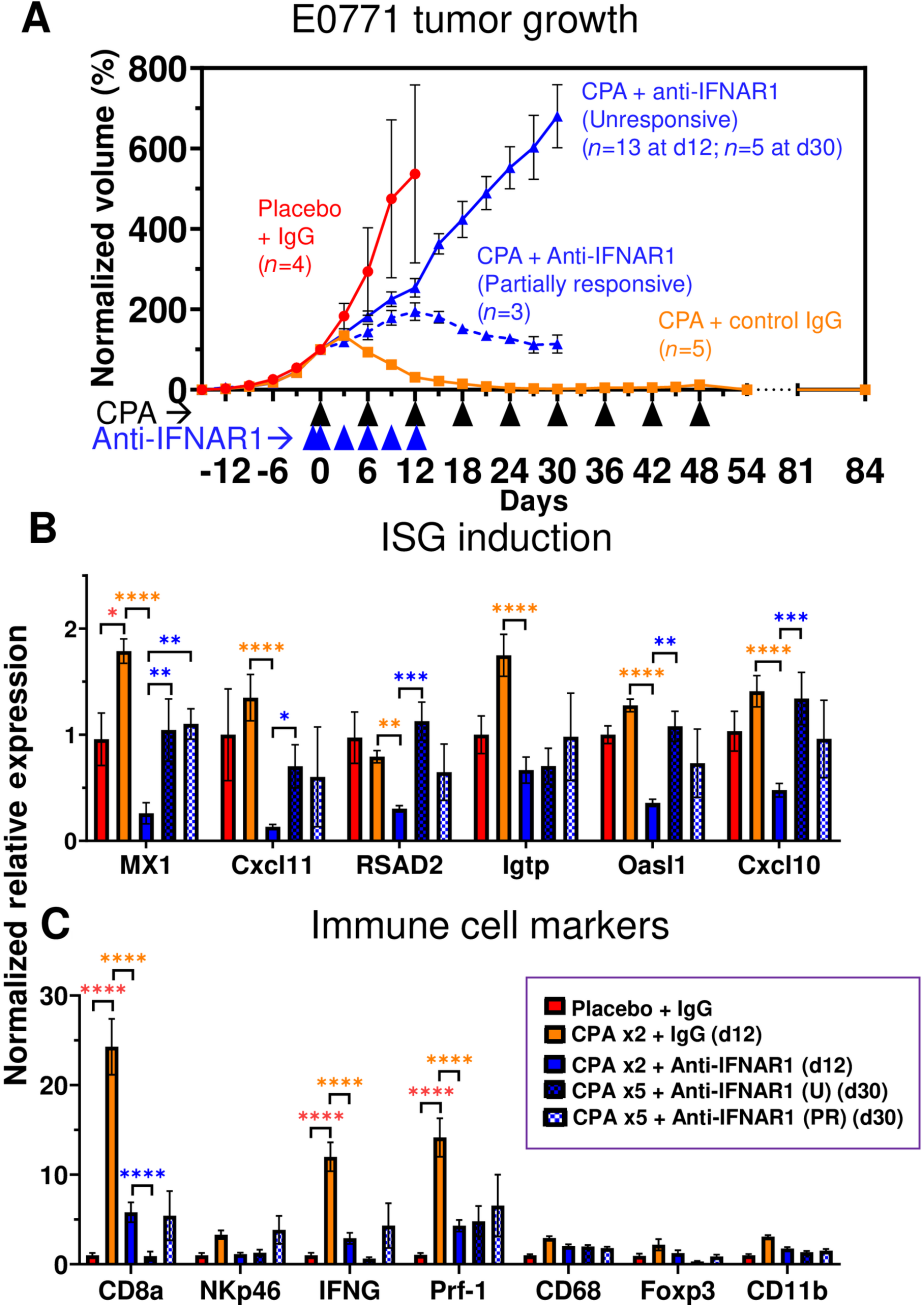
Type I IFN signaling is required for E0771 tumor regression. **A,** E0771 tumors were treated with metronomic CPA, as in Fig. 5, alone or in combination with anti-IFNAR1 to block type I IFN signaling. Data shown are mean ± SEM volumes for *n* = 4 tumors for the placebo + IgG group, *n* = 5 for CPA + control IgG, and *n* = 16 (through day 12) for CPA + anti-IFNAR1 (decreasing to *n* = 8 from day 12–30, due to 8 tumors excised for analysis on day 12). Tumors in the CPA + anti-CD8α group showed two distinct growth patterns: Unresponsive, with strong continued growth through day 30; and partially responsive, as indicated by growth stasis or moderate regression, as marked. Tumor volumes were normalized to the percent of day 0 volume ( = 100). Significance (two-way ANOVA): *, *P* < 0.05; **, *P* < 0.01; ***, *P* < 0.001; ****, *P* < 0.0001. **B** and **C,** Tumor ISGs were significantly depleted by anti-CD8α antibody, which also suppressed CPA-induced CD8 T-cell infiltration and IFNG and Prf-1 production. Of note, in this cohort of mice, the CPA + control IgG tumors were eradicated and by day 84 did not show the tumor regrowth seen in the mice shown in Fig. 7A.

We used an inhibitory IFNAR1 antibody to determine the functional role of type-I IFN signaling and the impact of the transient, downstream induction of ISGs on CPA-induced immune cell recruitment and tumor regression. Mice bearing E0771 tumors were given anti-mouse IFNAR1, or control IgG, beginning 1 day prior to the first CPA treatment on day 0 (Fig. 8A). Remarkably, the major tumor regression seen by day 12 in the CPA + control IgG mouse group was fully blocked in all 16 mice given CPA + anti-IFNAR1 antibody. Furthermore, robust tumor growth persisted in 5 of the 8 mice that we continued to monitor after anti-IFNAR1 treatment was halted on day 12, and only moderate regression was observed in the 3 other mice, despite ongoing CPA treatment. This result contrasts to the growth static response to CPA seen in CD8 T cell–depleted tumors (Fig. 7) and indicates that direct CPA tumor cell cytotoxicity has limited impact on tumor growth in the absence of IFNAR1 signaling.

Gene expression analysis showed that anti-IFNAR1 antibody reduced ISG expression below control tumor levels by day 12, indicating the antibody is highly effective in blocking tumor IFN signaling (Fig. 8B). ISG expression returned to basal levels by day 30, that is, 18 days after antibody treatment was halted on day 12. Anti-IFNAR1 also reduced tumor infiltration of all tested immune cells by day 12 (Fig. 8C). The reduction of tumor-infiltrating CD8 T cells was further supported by FACS analysis of tumor tissue (Supplementary Fig. S9A) and occurred without any changes in circulating CD8 T cells (Supplementary Fig. S9B). Thus, depletion of tumor-infiltrating CD8 T cells, and likely other infiltrating immune cells, is a consequence of the inhibition of tumor type I IFN signaling and not a systemic effect. Day 12 CD8α T-cell marker levels were restored by day 30 in the three anti-IFNAR1–treated tumors that were partially responsive to CPA, but not in the five CPA-unresponsive tumors (Fig. 8C, PR vs. U groups), which could help explain their differences in CPA responsiveness.

## Discussion

Effective treatment continues to be challenging, for many breast cancer patients, with limited therapeutic options and high rates of disease recurrence (43). Cytotoxic chemotherapies, including CPA, remain the primary systemic treatment modality for TNBC despite highly variable treatment responses and frequent development of chemo-resistance (44). Here, we explored the role of innate immunity in relation to cytotoxic treatment response and resistance. We used two orthotopic mouse models, 4T1 and E0771, to investigate the chemo-immunogenic activity of CPA when delivered on a metronomic, medium-dose intermittent (MEDIC) schedule (24). 4HC, a chemically activated CPA derivative that spontaneously decomposes to yield the same active metabolite as CPA, induced the expression of hundreds of ISGs in both cell models in a manner similar to doxorubicin, an established immune-stimulatory chemotherapeutic agent (45). These tumor cell-centric ISG responses to activated CPA were at least in part dependent on signaling by the type I IFN receptor, IFNAR1, implicating tumor cell production of type I IFNs in these drug-induced ISG responses. Many of the ISG responses seen in cell culture were recapitulated *in vivo* in MEDIC CPA-treated tumors implanted in syngeneic mice. Notably, CPA-treated 4T1 tumors showed robust type I IFN signaling and tumor immune cell infiltration, leading to an overall tumor growth static response. In contrast, E0771 tumors exhibited a somewhat weaker IFN response, but this was followed by robust immune infiltration and extensive tumor regression, both of which were absolutely dependent on type-I IFN signaling by IFNAR1. Thus, a robust IFN-mediated immune response may be essential for the efficacy of metronomic CPA, at least in some forms of breast cancer. Furthermore, our findings raise the possibility that treatment resistance to CPA, and perhaps other chemo-immunogenic cytotoxic agents, may stem from silencing of the IFN pathway.

The 4T1 cell line is generally considered to share many characteristics of TNBC at the genomic and transcriptomic levels (33) but there is disagreement about the molecular subtype of the E0771 cell line, with prior literature suggesting it is either representative of TNBC or of a luminal (estrogen receptor positive) subtype (46, 47). For example, a recent study measuring receptor expression (RNA) and protein levels reported that E0771 cells are estrogen receptor-α negative, estrogen receptor-β positive, progesterone receptor positive, and Her2 (Erbb2) positive, that is, have a luminal B molecular subtype (47). In contrast, analysis of the E0771 cells used in this study revealed that our E0771 cells were estrogen receptor-α negative, estrogen receptor-β negative, progesterone receptor negative, and Her2 (Erbb2) positive. These results were confirmed in both untreated and drug-treated E0771 cells in cell culture, as well as in E0771 tumors implanted in syngeneic mice (Supplementary Fig. S1); thus, the E0771 cells used in this work do not represent a luminal subtype. It is not clear what explains the difference between our findings and those reported in (47), but presumably it relates to subline differences, which are apparently widespread for E0771 cells (46). In contrast, the 4T1 cells used here had a hormone receptor expression pattern consistent with prior work (33), namely, high expression of Erbb2, moderate to low expression of estrogen receptor α, and no expression of progesterone receptor, a pattern typically considered to be representative of TNBC.

ISG induction was an early response to drug treatment in both breast cancer models, both in cell culture and following CPA treatment of implanted tumors *in vivo*. The ISG responses seen *in vivo* were transient (Fig. 6B) and were followed by strong increases in both innate and adaptive infiltrating immune cells, including NK cells and CD8α T cells, after 6–12 days (i.e., 1–2 CPA treatment cycles). Type I IFNs and the ISGs they induce are known to stimulate T cells, NK cells, macrophages and dendritic cells, and other immune cells (20). ISG induction may thus be a useful marker for immunogenic potential *in vivo*. Of note, the ISG responses seen in our cell models were not apparent until 48 hours after drug treatment, even though ISG gene induction *per se* is a rapid process, as was seen when the cells were treated with IFNβ directly (Supplementary Fig. S5). The delay in ISG induction seen in CPA-treated cells and tumors likely reflects time required for CPA to effect tumor cell damage and the associated production of immunostimulatory damage-associated molecular pattern molecules. These may include dsRNAs and nucleic acid agonists of STING, which can activate cytosolic sensors and induce type I IFN production through established mechanisms (23).

Using RNA-seq, we validated the transient nature of CPA-induced ISG responses on a global scale. We identified a set of 188 ISGs that responded in common to IFNβ and to 4HC treatment in cultured E0771 cells, as well as 380 commonly responding ISGs in 4T1 cells. Strikingly, 52 of the 188 E0771 ISGs showed an early, transient response in CPA-treated E0771 tumors, where they comprised 71% of the early-transient response gene set, representing a 61-fold enrichment compared with a background set of all expressed genes (Fig. 6). These 52 genes comprise a robust set of CPA-responsive E0771 ISGs and could serve as useful markers for early chemo-immunogenic responses to CPA treatment *in vivo*.

The ability of anti-IFNAR1 antibody to almost completely abolish CPA-induced E0771 tumor regression establishes that type I IFN signaling is essential for the anti-tumor actions of metronomic CPA in this model. This, in turn, leads us to the unexpected conclusion that the intrinsic tumor cell cytotoxicity of CPA does not translate into a major therapeutic response in the absence of type I IFN signaling. These findings support a model whereby CPA-induced tumor cell damage induces the major antitumor effects of CPA on E0771 tumors indirectly, via its ability to activate tumor cell autonomous type I IFN signaling linked to an immunogenic cell death mechanism. Of note, we observed tumor growth stasis when circulating and tumor cell infiltrating CD8 T cells were immunodepleted, that is, the block in CPA antitumor activity was less complete than with anti-IFNAR1 antibody. This tumor growth stasis response is likely mediated by other tumor-infiltrating immune cells, for example, NK cells, whose CPA-induced levels were further increased by CD8α T-cell depletion (Fig. 7C). The ability of NK cells to contribute to the anti-tumor effects of metronomic CPA is supported by earlier findings in glioma models (26, 48). Further study will be required to investigate important questions concerning the downstream mechanisms whereby the immune cell activation induced in MEDIC CPA-treated mammary tumors leads to the robust tumor regression observed in the E0771 tumor model, including whether tertiary lymphoid structures are involved (49).

The two models studied here, 4T1 and E0771, exhibited notable differences in their ISG, immune cell, and therapeutic responses to MEDIC CPA treatment *in vivo*. 4T1 tumors were characterized by a stronger and longer lasting ISG induction, but this did not translate into a greater anti-tumor response. This is evidenced by the growth stasis observed in 4T1 tumors versus the major regression seen in E0771 tumors. Combination chemo-immuno therapies designed to stimulate immunogenic cell death and activate a more robust antitumor response (50) may be required for more effective treatment of 4T1 tumors. Differences in mouse strain, tumor cell proliferation and angiogenesis, and mutational burden, which is much higher in E0771 than 4T1 tumors (51), could contribute to the differential responsiveness of these two models to CPA treatment. In addition, immunosuppressive regulatory T cells (Foxp3^+^ CD4^+^) increase with time in both models, but only E0771 tumors display an early growth period when a majority of tumor-associated CD4^+^ T cells are immunostimulatory (31), resulting in a more favorable environment for CPA responses. Further study of the mechanisms underlying metronomic CPA-induced E0771 tumor regression and the comparative resistance of 4T1 tumors may help identify useful biomarkers for tumor responsiveness and could lead to the discovery of new molecular targets for increasing effectiveness of chemotherapy in poorly responsive breast cancer. Recent clinical trials (52–56) as well as preclinical studies (57, 58) have used CPA in combination with other drugs to treat breast cancer with varying degrees of success and there may be opportunities for further improvements based on MEDIC scheduling once dose and schedule are optimized in the clinic (24). Furthermore, it will be important to determine whether a 7-day metronomic schedule can be as effective as the 6-day MEDIC schedule used here and in our prior preclinical studies in glioma models (19, 26, 27, 48), given the practical difficulties that are likely to be encountered with introducing a 6-day treatment schedule into the clinic. Improvements based on combination with checkpoint inhibitors are also possible, as suggested by the increased expression following MEDIC cyclophosphamide treatment of both PD-1 and PD-L1 reported for other murine breast cancer models (28) and seen here in both E0771 and 4T1 tumors (Supplementary Fig. S1).

Finally, the treatment models developed here may provide an important means to develop clinically translatable markers of chemo-immunogenic treatment response and resistance. The gene signatures identified by our RNA-seq analysis may be useful in pretreatment biopsies to identify individual patients most likely to elicit an IFN-mediated treatment response. There is also substantial interest in identifying noninvasive imaging markers of chemotherapy treatment response (59). Therapy-induced responses, including apoptosis, proliferation, and overall treatment response, can be monitored in real time in preclinical oncology models by label-free optical imaging techniques, such as spatial frequency domain imaging (60, 61).The preclinical therapy models described here provide a means to discover novel imaging markers that discriminate chemo-immunogenic sensitive and resistance tumors (62). As similar imaging markers can be tracked in patients with clinical imaging modalities such as PET and optical diffuse optical spectroscopy, it may be possible to rapidly identify both treatment response and resistance and adjust the treatment regimen accordingly (63, 64).

## Supplementary Material

Supplementary Figures S1-S9 Figures S1-S9 with detailed legends:
Fig. S1. Hormone receptor and related gene expression patterns in E0771 and 4T1 tumor cells. (A) Expression in E0771 cells and E0771 tumors, based on RNAseq data included in Table S1 and Table S5; and (B) Expression in 4T1 cells and 4T1 tumors.(C) Summary of hormone receptor expression patterns.
Fig. S2. Gene-specific qPCR primer sequences, amplicon length and percent GC content
Fig. S3. Dose-dependence of drug sensitivity of cultured 4T1, E0771 and B16F10 cells. Shown are viability assays determined in MTS assays.
Fig. S4. ISG induction by 4HC-conditioned culture medium.
Fig. S5. Verification of anti-IFNAR-1 antibody inhibitory activity.
Fig. S6. E0771 tumor growth curves and qPCR analysis of metronomic-CPA in vivo dose response data.
Fig. S7. Representative FACS analysis of blood from CPA-treated mice, with and without anti-CD8a antibody treatment.
Fig. S8. Circulating CD8 T-cells for CPA-treated mice with and without anti-CD8a antibody.
Fig. S9. FACS analysis of blood and tumors from mice given metronomic CPA treatment with and without anti-IFNAR1 antibody.

Table S1 Table S1. RNAseq analysis of cultured 4T1 and E0771 tumor cell RNA.

Table S2 Table S2. Results of DAVID analysis showing output of Functional Annotation Clustering for all annotation clusters with an overall cluster enrichment score > 1.3.

Table S3 Table S3. RNAseq library sequencing stats.

Table S4 Table S4. RNAseq analysis of 4T1 tumors, with vs without metronomic CPA treatment.

Table S5 Table S5. RNAseq analysis of E0771 tumors, with vs without CPA treatment.
