# Supplementary material for: Type-I Interferon Signaling Is Essential for Robust Metronomic Chemo-Immunogenic Tumor Regression in Murine Breast Cancer
Source: Cancer Res Commun. 2022 Apr 22;2(4):246–57. doi: 10.1158/2767-9764.CRC-21-0148 (PMC9524291; doi:10.1158/2767-9764.CRC-21-0148)
Supplement: Supplementary Figures S1-S9 — Figures S1-S9 with detailed legends: Fig. S1. Hormone receptor and related gene expression patterns in E0771 and 4T1 tumor cells. (A) Expression in E0771 cells and E0771 tumors, based on RNAseq data included in Table S1 and Table S5; and (B) Expression in 4T1 cells and 4T1 tumors.(C) Summary of hormone receptor expression patterns. Fig. S2. Gene-specific qPCR primer sequences, amplicon length and percent GC content Fig. S3. Dose-dependence of drug sensitivity of cultured 4T1, E0771 and B16F10 cells. Shown are viability assays determined in MTS assays. Fig. S4. ISG induction by 4HC-conditioned culture medium. Fig. S5. Verification of anti-IFNAR-1 antibody inhibitory activity. Fig. S6. E0771 tumor growth curves and qPCR analysis of metronomic-CPA in vivo dose response data. Fig. S7. Representative FACS analysis of blood from CPA-treated mice, with and without anti-CD8a antibody treatment. Fig. S8. Circulating CD8 T-cells for CPA-treated mice with and without anti-CD8a antibody. Fig. S9. FACS analysis of blood and tumors from mice given metronomic CPA treatment with and without anti-IFNAR1 antibody. [file crc-21-0148-s01.pdf]

Fig. S1AB

A. E0771			E0771 cells	E0771 cells + 4HC (72 hr)	E0771 cells + IFNbeta (6 hr)	E0771 tumors	E0771 tumors + CPA (2 cycles)
Gene ID	Gene Descriptor	**E0771 expression** [Le Naour et al, Can Med 2020 review]	798: Intensity-1	798: Intensity-2	799: Intensity-2	704: Intensity-1	708: Intensity-2
ERBB2	HER2 (EGF receptor-2)	Positive/Negative	4.71	3.41	2.70	3.58	1.49
ESR1	ER-alpha	Positive/Negative	0.01	0.05	0.04	0.06	0.54
ESR2	ER-beta	Negative	0.00	0.01	0.01	0.77	0.50
PGR	Progesterone receptor	Negative	0.00	0.00	0.00	0.00	0.19
HRH1	Histamine receptor	Negative	0.05	0.07	0.11	0.00	0.00
HRH2	Histamine receptor	Negative	0.00	0.04	0.01	0.07	0.20
KRT5	Cytokeratin	Negative	0.02	0.05	0.03	0.12	0.25
NTRK1	Trk/BDNF oncogenic pathway	Negative	0.02	0.08	0.07	0.02	0.92
NTRK3	Trk/BDNF oncogenic pathway	Negative	0.00	0.01	0.01	0.04	0.06
PLAC1	Breast cancer biomarker	Positive	0.80	0.61	0.58	0.01	0.22
CLDN1	Claudin family	Positive	0.00	0.00	0.00	0.17	0.03
CLDN10	Claudin family	Positive	0.02	0.01	0.04	0.02	0.21
CLDN7	Claudin family	Positive	0.00	0.00	0.01	0.04	0.28
EGFR	EGF receptor	Positive/Negative	0.80	0.96	0.78	0.05	1.33
DLL4	Notch ligand	Positive	0.01	0.02	0.02	1.43	2.11
JAG1	Notch ligand	Positive	1.55	2.32	2.23	1.80	2.71
NOTCH2	Notch receptor	Positive	10.67	10.76	8.59	8.14	5.26
NOTCH3	Notch receptor	Positive	0.00	0.02	0.02	0.19	0.51
NOTCH4	Notch receptor	Positive	0.09	0.20	0.18	3.04	3.08
PDCD1	PD1, immune modulator	Positive	0.00	0.02	0.01	0.62	5.76
CD274	PDL1, immune modulator	Positive	0.25	1.94	0.78	3.86	15.32
NTRK2	Trk/BDNF oncogenic pathway	Positive	0.00	0.01	0.01	0.04	1.57

B. 4T1			4T1 cells	4T1 cells + 4HC (72 hr)	4T1 cells + IFNbeta (6 hr)	4T1 tumors	4T1 tumors + CPA (2 cycles)
Gene ID	Gene Descriptor	**E0771 expression** [Le Naour et al, Can Med 2020 review]	882: Intensity-1	882: Intensity-2	885: Intensity-2	700: Intensity-1	700: Intensity-2
ERBB2	HER2 (EGF receptor-2)	Positive/Negative	3.43	3.76	5.98	6.94	6.13
ESR1	ER-alpha	Positive/Negative	0.89	0.75	0.39	1.31	1.16
ESR2	ER-beta	Negative	0.22	0.63	0.19	1.13	1.04
PGR	Progesterone receptor	Negative	0.00	0.00	0.00	0.06	0.14
HRH1	Histamine receptor	Negative	0.03	0.18	0.06	0.06	0.06
HRH2	Histamine receptor	Negative	0.03	0.14	0.01	0.16	0.23
KRT5	Cytokeratin	Negative	0.00	0.01	0.01	0.17	0.32
NTRK1	Trk/BDNF oncogenic pathway	Negative	0.00	0.01	0.00	0.35	6.99
NTRK3	Trk/BDNF oncogenic pathway	Negative	0.00	0.00	0.00	0.07	0.06
PLAC1	Breast cancer biomarker	Positive	0.00	0.00	0.00	0.03	0.05
CLDN1	Claudin family	Positive	0.19	0.84	0.13	0.69	0.41
CLDN10	Claudin family	Positive	0.01	1.31	0.07	0.12	0.24
CLDN7	Claudin family	Positive	0.00	0.00	0.00	0.11	0.17
EGFR	EGF receptor	Positive/Negative	15.26	16.83	19.51	11.41	10.08
DLL4	Notch ligand	Positive	0.25	1.56	0.18	3.34	2.97
JAG1	Notch ligand	Positive	3.59	7.55	3.34	5.39	4.95
NOTCH2	Notch receptor	Positive	5.79	7.59	6.81	10.46	10.72
NOTCH3	Notch receptor	Positive	0.01	0.05	0.01	1.41	1.70
NOTCH4	Notch receptor	Positive	0.01	0.12	0.02	3.10	3.27
PDCD1	PD1, immune modulator	Positive	0.00	0.00	0.00	0.65	2.26
CD274	PDL1, immune modulator	Positive	1.00	1.71	4.39	3.85	10.58
NTRK2	Trk/BDNF oncogenic pathway	Positive	0.00	0.00	0.00	0.19	0.11

Fig. S1C

C. Summary				
	**ER-alpha**	**ER-beta**	**Progest receptor**	**HER2**
**Luminal A**	Positive	*Not specified*	Pos/Neg	Negative
**Luminal B**	Positive	*Not specified*	Pos/Neg	Positive
**Triple negative**	Negative	*Not specified*	Negative	Negative
**HER2+**	Negative	*Not specified*	Negative	Positive
**E0771** (Can Med 2020, literat. review)	Pos/Neg	Negative	Negative	Pos/Neg
**E0771** (Le Naour, 2020)	Negative	Positive	Positive	Positive
**E0771 cells** (this study)	Negative	Negative	Negative	Positive
**E0771 tumors** (this study)	Negative	Positive	Negative	Positive

Fig. S1. Hormone receptor and related gene expression patterns in E0771 and 4T1 tumor cells. (**A**) Expression in E0771 cells and E0771 tumors, based on RNAseq data included in Table S1 and Table S5; and (**B**) Expression in 4T1 cells and 4T1 tumors, based on RNAseq data included in Table S1 and Table S4. Gene expression intensity values are in FPKM (fragments per kb per million reads). Genes listed in A and B are from Table 3 of Le Naour *et al*, Cancer Medicine (2020) PMID: 33026171. The third column lists whether the gene is expressed or not expressed in E0771 cells based on a literature review carried out by the authors of that publication. Positive/Negative indicates that different studies reported opposite results for that gene. (**C**) Summary of hormone receptor expression patterns. The top four rows indicate the expected expression patterns for each of the indicated four “intrinsic” molecular subtypes of breast cancer, as shown in Table 1 of Le Naour *et al*, Canc Cell Int (2020), PMID: 32699527, while the bottom 4 rows summarize results for E0771 cells and tumors, as indicated.

Fig. S2: Gene-specific qPCR primer sequences, amplicon length and % GC content

ON#	Gene	Direction	Sequence (5'-3')	Amplicon Length (bp)	G:C Content (%)
6987	Mx1	Forward	AGAGCTCTGTGCTGGAAGCAC	93	57
6988		Reverse	GCTTCCTCAATTTCAGCACCA	93	48
7484	Oasl1	Forward	GATGTGCGCGTGCTCAAG	80	61
7485		Reverse	CACGGTGCCATTCCCAAA	80	56
3373	Cxcl10	Forward	ACCATGAACCCAAGTGCTGCC	140	57
3374		Reverse	CTATGGCCCTCATTCTCACTGGCC	140	58
6955	Igtp	Forward	CTGAGCCTGGATTGCAGCTT	81	55
6956		Reverse	TGGGTCTGCTCTAGGCCTTG	81	60
8065	RSAD2	Forward	GCCCAAGTATTCACCCCTGT	133	55
8066		Reverse	AAGACATCCTTCGTGCTGCC	133	55
7936	Cxcl11	Forward	ACGGCTGCGACAAAGTTGAA	95	50
7937		Reverse	GGAGGGCTCACAGTCAGACG	95	65
4255	CD8α	Forward	GAAGATTCTGGGGCAGCATGGCAAAG	81	54
4256		Reverse	TTGGAATCAAAACGATCAA	81	30
4668	Nkp46	Forward	GCAACCCCCTGAAACTGGTA	79	55
4669		Reverse	AAGGTTACCTCAGGCTGTGGATA	79	48
3427	CD68	Forward	GCCCGAGTACAGTCTACCTGG	97	62
3428		Reverse	GCCCGAGTACAGTCTACCTGG	97	45
4253	Foxp3	Forward	GCCTTCAGACGAGACTTGGAA	99	52
4254		Reverse	CTGGCCTAGGGTTGGGCATT	99	60
4261	CD11b	Forward	CCAAGAGAATGCAAAAGGCTTT	74	41
4262		Reverse	GGGGGGCTGCAACAACCACA	74	65
3429	IFNG	Forward	TCTTCAGCAACAGCAAGGCG	79	55
3430		Reverse	CGCTGGACCTGTGGGTTGTTG	79	62
3565	Prf-1	Forward	GTACAACTTTAATAGCGACACAGTA	80	36
3566		Reverse	AGTCAAGGTGGAGTGGAGGT	80	55
3561	GZMB	Forward	TGTCTCTGGCCTCCAGGACAA	110	57
3562		Reverse	CTCAGGCTGCTGATCCTTGATCGA	110	54

Fig. S2. Gene-specific qPCR primer sequences, amplicon length and percent GC content

Fig. S3

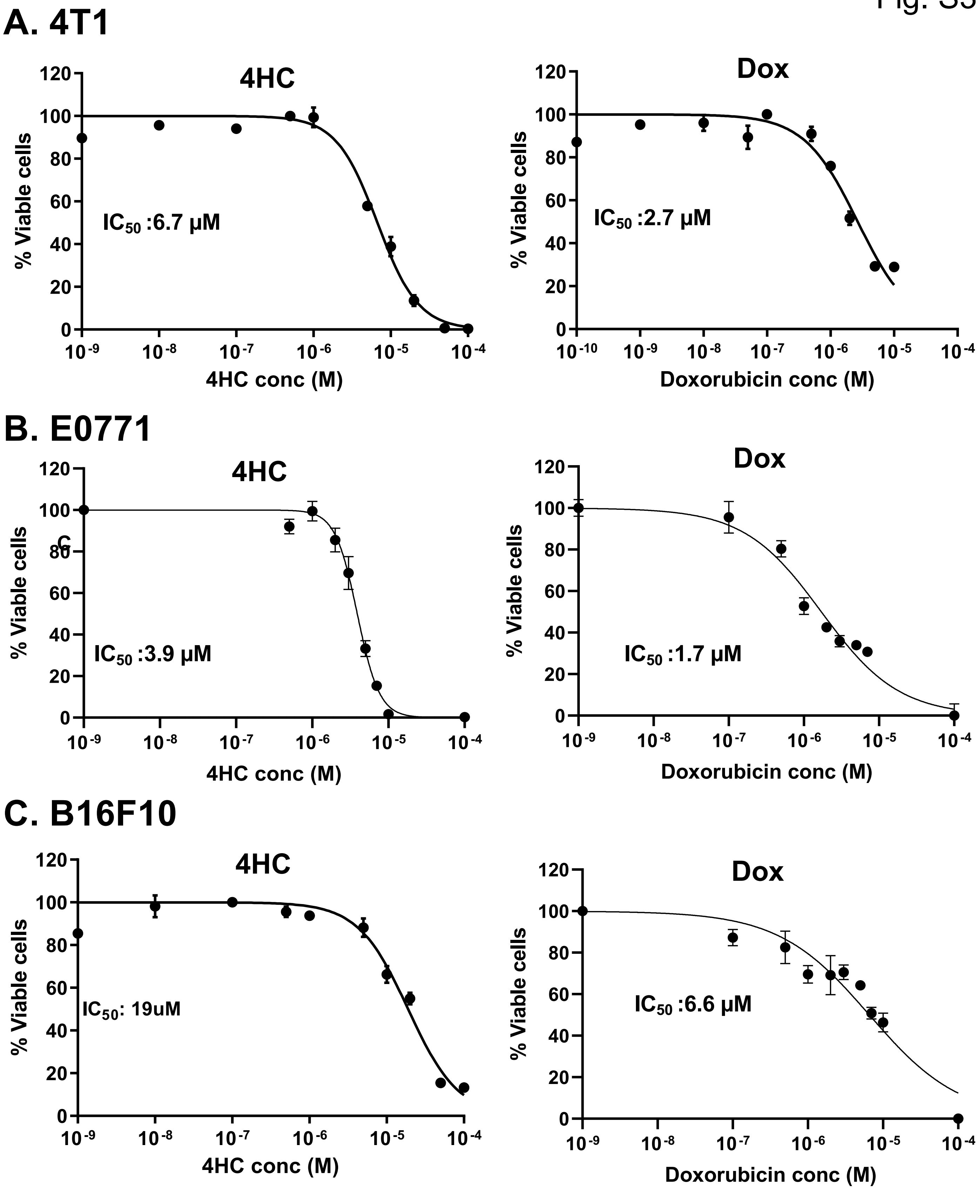

Fig. S3. **Dose-dependence of drug sensitivity of cultured 4T1, E0771 and B16F10 cells**. Shown are viability assays determined in MTS assays over multi-$\log_{10}$ range of 4HC (left) and doxorubicin (right) for 4T1 (**A**), E0771 (**B**) and B16F10 cells (**C**). Data points: mean +/- SD values for n = 3 wells of a 96-well plate. IC50 values were determined using log (inhibitor) vs normalized response function in GraphPad Prism.

Fig. S4: ISG induction by 4HC-conditioned media

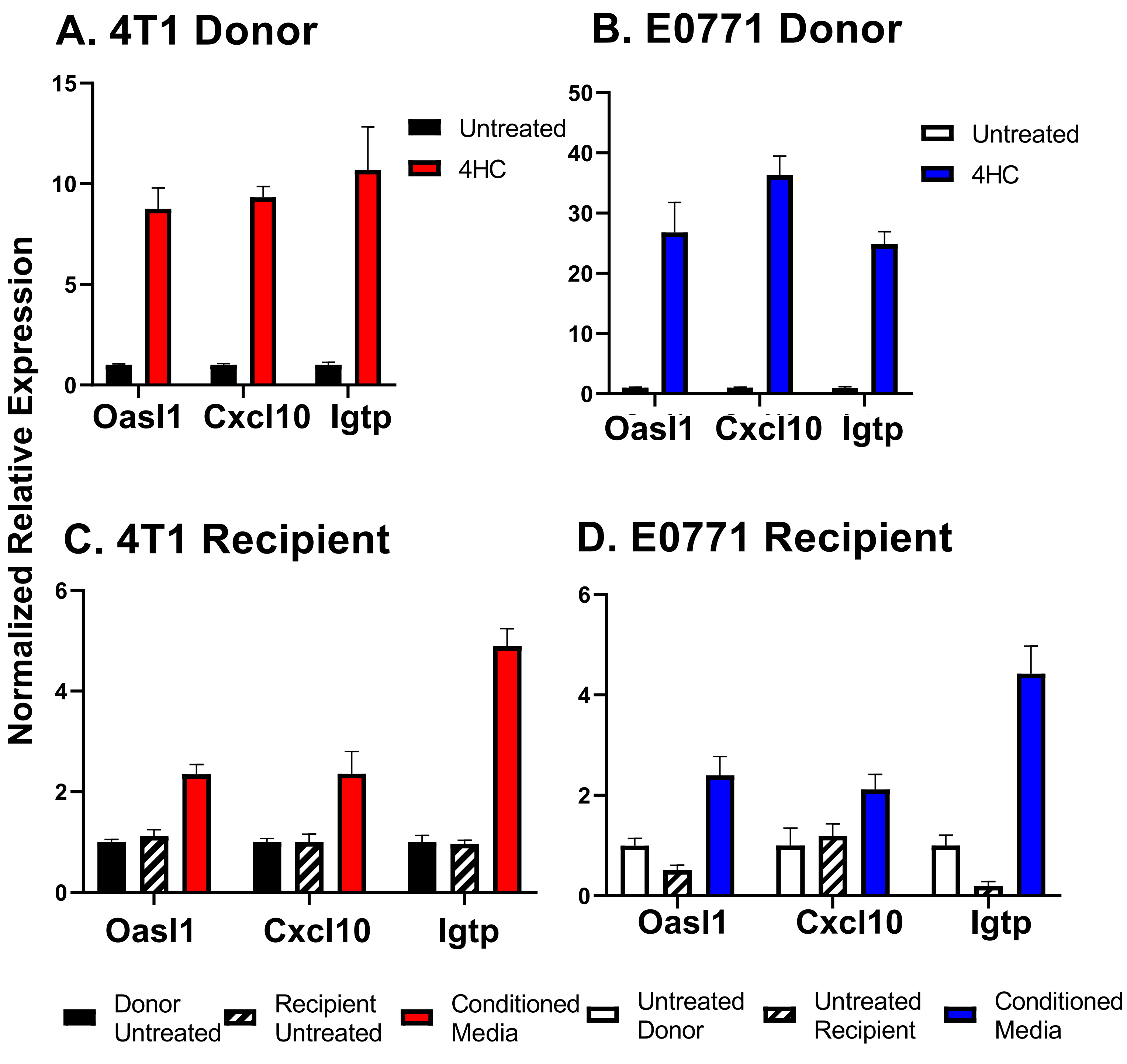

Fig. S4. ISG induction by 4HC-conditioned culture medium. **A, B**. 4T1 and E0771 cells were treated with 4HC for 72-h under the same conditions as Fig. 1. Data show ISGs were strongly induced in both cell models, as determined by qPCR analysis. **C, D**. Induction of ISGs in drug-naïve 4T1 and E0771 recipient cells treated for 4-h with 4HC-conditioned cell culture supernatant from the corresponding drug-treated donor cells (as in A, B), followed by a PBS wash and 2 h incubation in fresh culture medium. Gene expression was analyzed by qPCR. In both cell lines, recipient cells showed weaker ISG induction than in cells directly exposed to 4HC. Data points: mean +/- SD values for n = 2-3 replicates representative of two independent experiments.

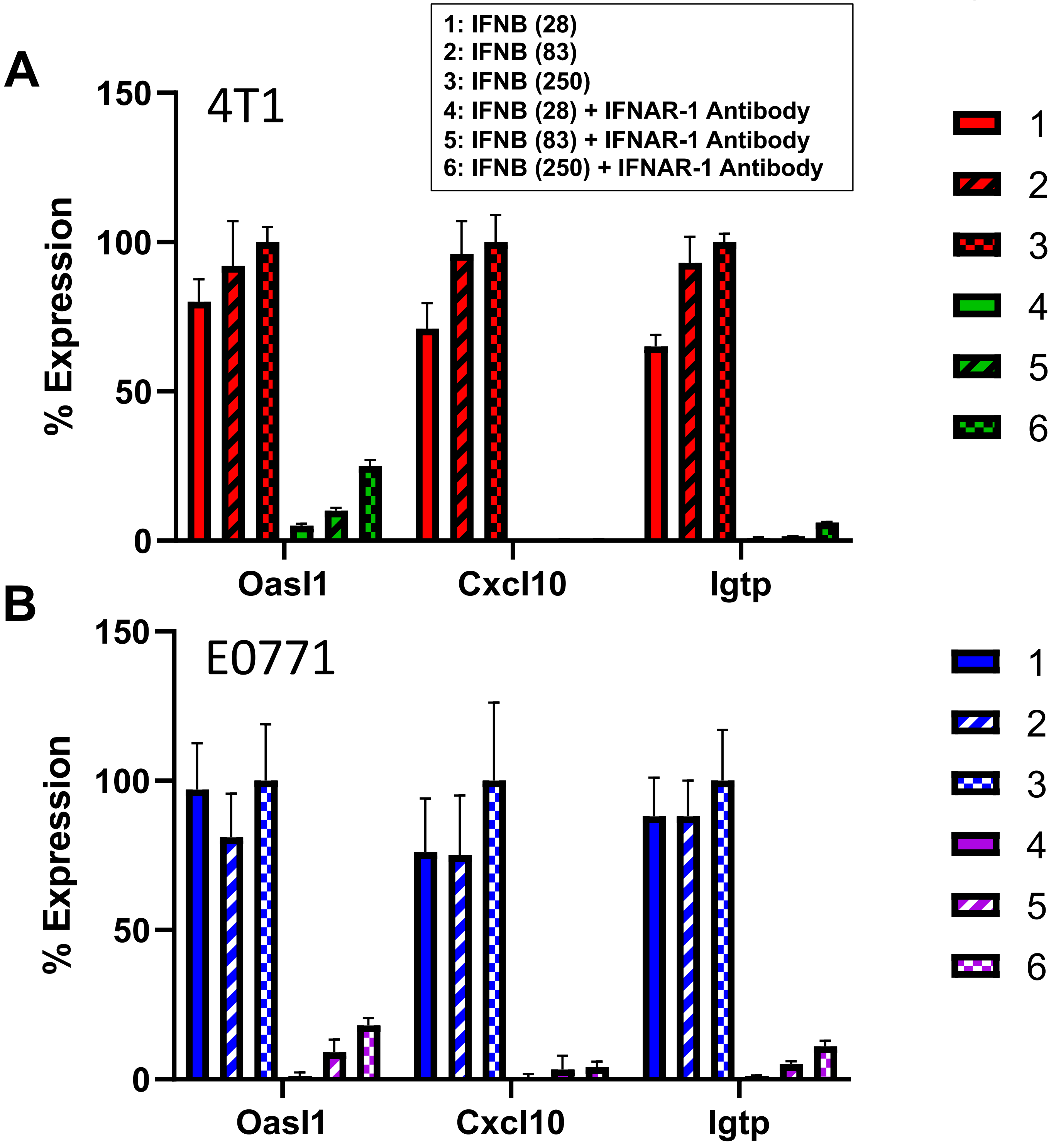

Fig. S5. Verification of anti-IFNAR-1 antibody inhibitory activity.
4T1 cells (**A**) and E0771 cells (**B**) were treated with 28, 83 or 250 U/mL of mouse recombinant IFNβ for 4-h, with or without 10 μg/mL IFNAR-1 antibody and harvested 2 h later. IFNβ induced similar ISG responses in both cell lines at all concentrations. Anti-IFNAR1 antibody blocked ISG induction > 90%, except at the highest concentration IFNβ for the ISG *Oasl1*, where the antibody concentration may not have been sufficient to effect complete inhibition. Data presented as mean +/- SD with n=3 replicates. Percent expression was calculated using the formula ((x ± SD) - (z ± SD)) / ((y ± SD) - (z ± SD)), where x = antibody + IFNβ treatment gene expression, z = untreated gene expression and y = IFNβ treatment gene expression.

Fig. S6

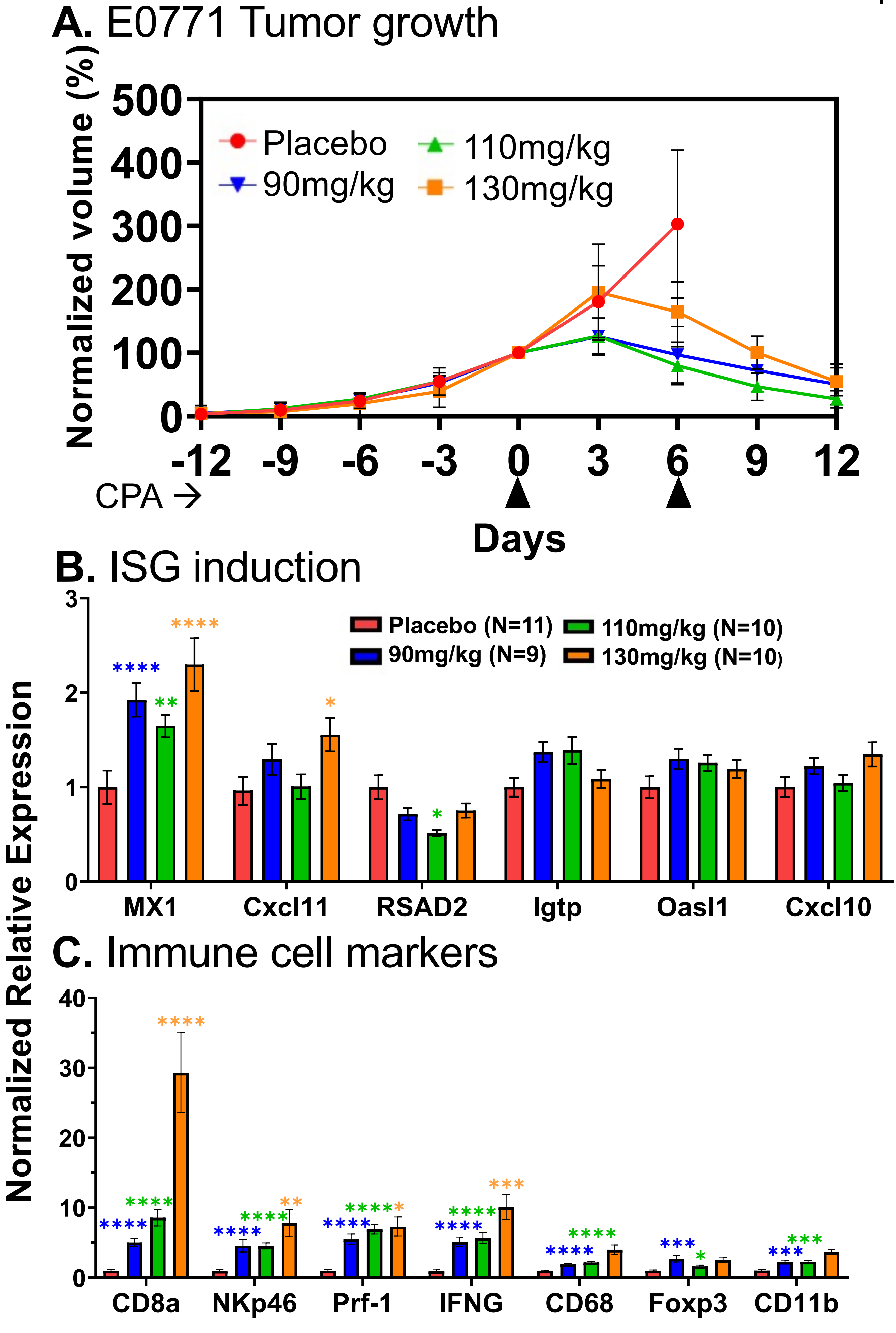

A. E0771 Tumor growth
Normalized volume (%)
500
400
300
200
100
0
Placebo
90mg/kg
110mg/kg
130mg/kg
-12
-9
-6
-3
0
3
6
9
12
CPA →
Days
B. ISG induction
Placebo (N=11)
90mg/kg (N=9)
110mg/kg (N=10)
130mg/kg (N=10)
3
2
1
0
MX1
Cxcl11
RSAD2
Igtp
Oasl1
Cxcl10
C. Immune cell markers
40
30
20
10
0
CD8a
NKp46
Prf-1
IFNG
CD68
Foxp3
CD11b
Normalized Relative Expression

Fig. S6. E0771 tumor growth curves and qPCR analysis of metronomic-CPA *in vivo* dose response data. **A**. E0771 tumors implanted in mice were treated with 90, 110 or 130 mg/kg CPA every 6 days. All three CPA dosages induced extensive tumor regression by day 12. Data shown are mean +/- SEM values for n = 11 tumors for the placebo group, n = 9 tumors for the 90 mg/kg CPA group, n = 10 tumors for the 110 mg/kg CPA group, and n = 10 tumors for the 130 mg/kg CPA group. Tumor volumes were normalized to 100 percent of the volume on Day 0 (first day of CPA treatment). **B**. ISG induction was < 2-fold at all CPA doses. **C**. Immune cell marker genes showed very similar fold-change values at each CPA dose, except for CD8a, which showed dose-dependent induction. Significance was determined by 2-way ANOVA: *, $p < 0.05$; **, $p < 0.01$; ***, $p < 0.001$; ****, $p < 0.0001$.

Fig. S7: Representative Anti-CD8a FACS Data

A. Size Selection

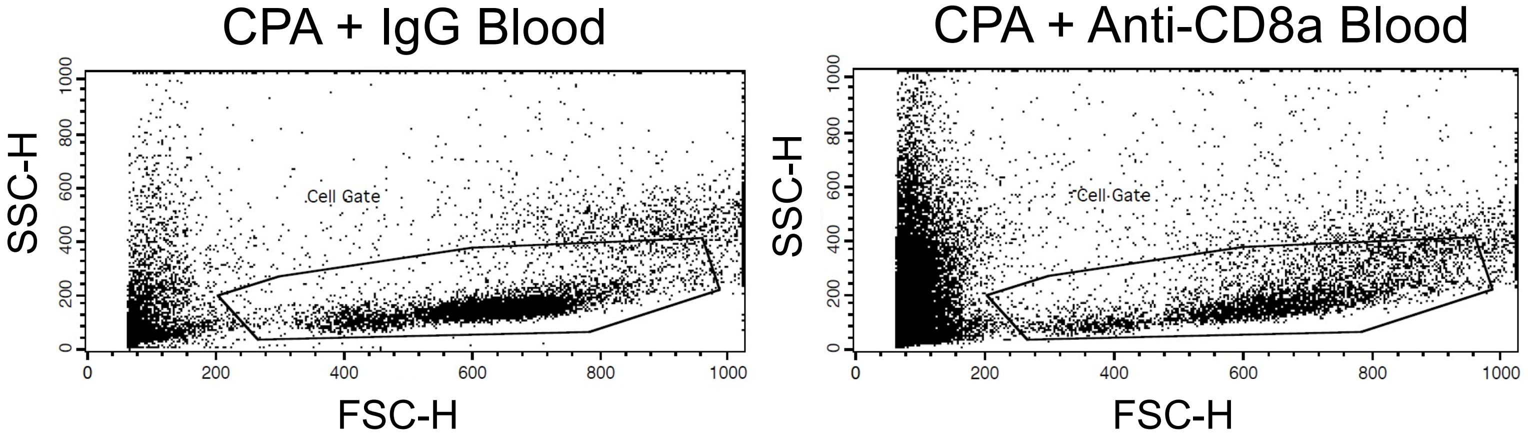

B. Dead Cell Exclusion:

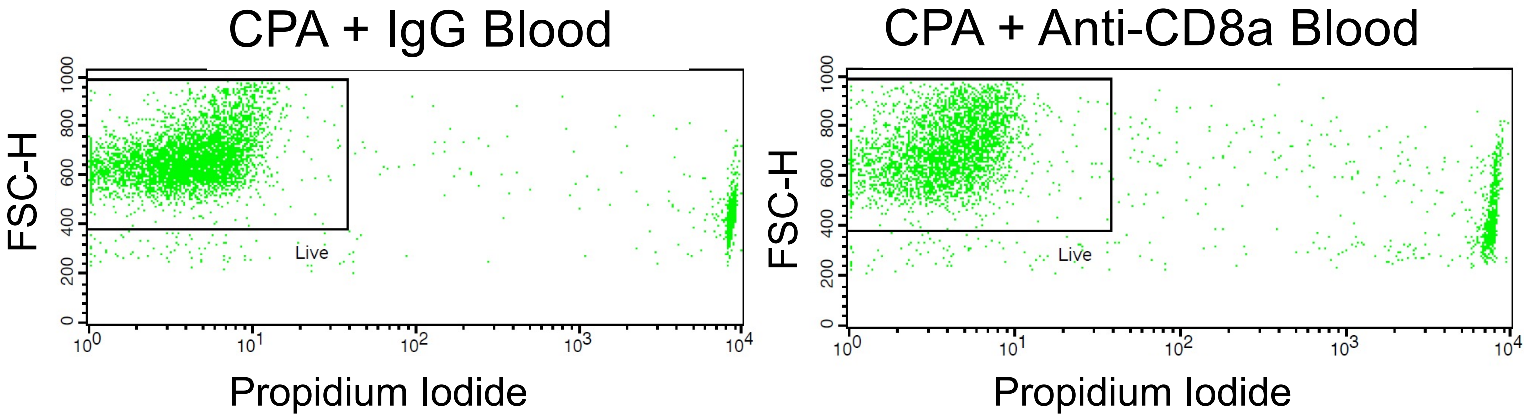

C. CD8a Selection:

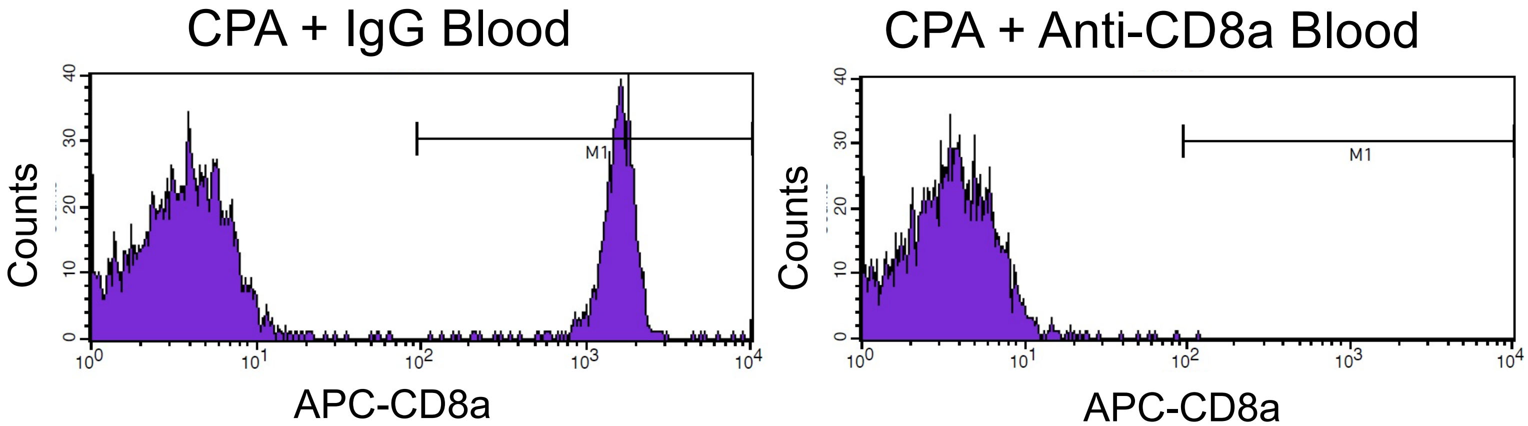

D. Histogram Statistics:

CPA + IgG Blood

Marker	Left, Right	Events	% Gated
All	1, 9910	5792	100.00
M1	95, 9910	1617	27.92

CPA + Anti-CD8a Blood

Marker	Left, Right	Events	% Gated
All	1, 9910	4115	100.00
M1	95, 9910	1	0.02

Fig. S7. Representative FACS analysis of blood from CPA-treated mice, with and without anti-CD8a antibody treatment. Data representative of 2 individual mice. **A**. 20 uL of mouse tail vein blood was prepared for FACS analysis of circulating CD8 T-cells. Events were selected based on general size parameters of forward-scatter (FSC-H) and side-scatter (SSC-H) to exclude overly large and small events. **B**. Live cells were selected by excluding events with propidium iodide signal. **C**. CD8 T-cells were selected by excluding events that lacked the APC signal from the APC-labeled anti-CD8a antibody used in sample preparation. **D**. CD8 T-cell percentages were calculated by dividing the CD8+ events by the total number of live events. Mouse blood from the CPA + anti-CD8a group was devoid of CD8 T-cells, in contrast to blood from the CPA + IgG control group.

Fig. S8: FACS Analysis during Metro-CPA + Anti-CD8a Antibody Treatment

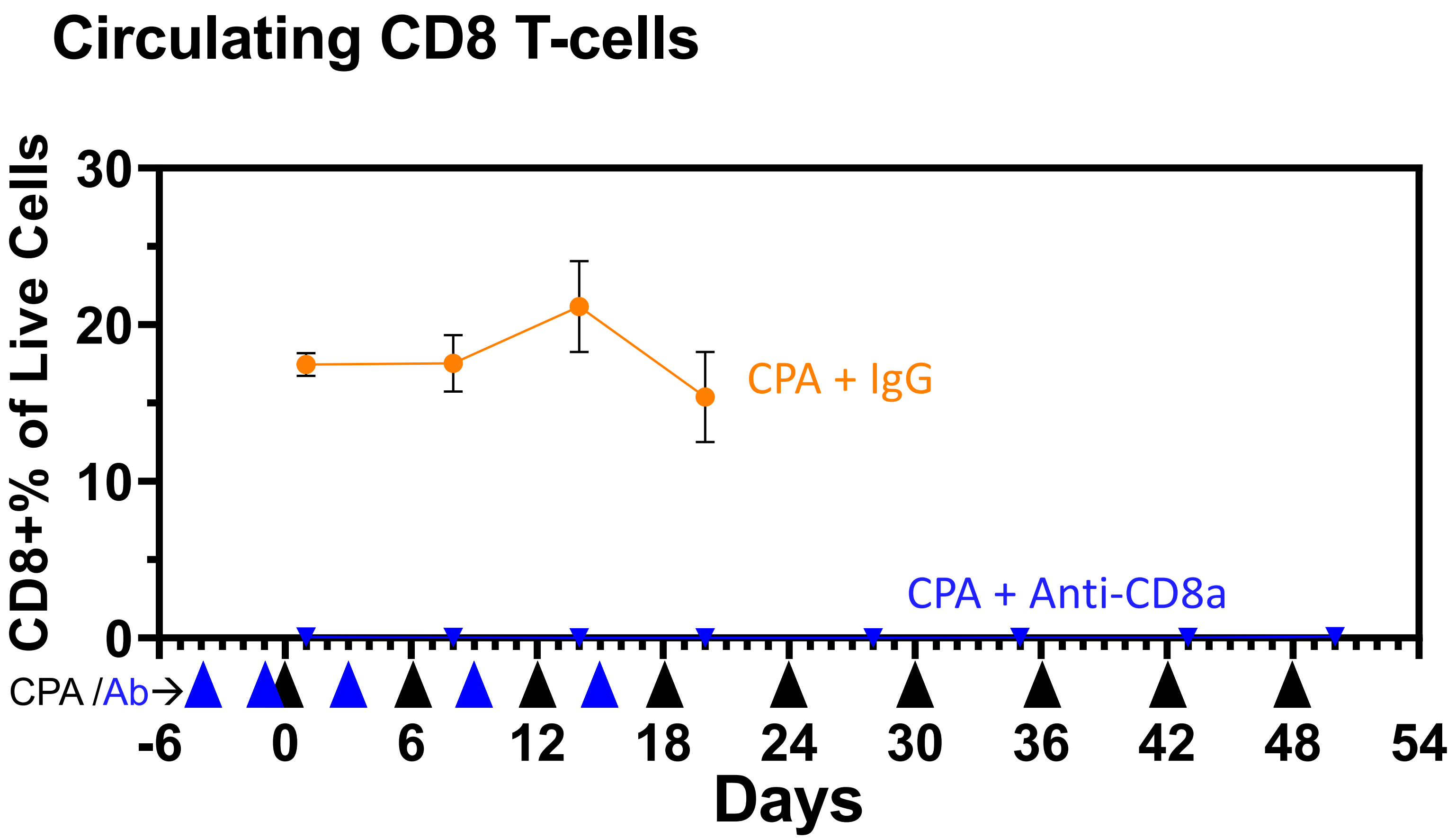

Fig. S8. Circulating CD8 T-cells for CPA-treated mice with and without anti-CD8a antibody. FACS analysis of blood from the mice shown in Fig. 7 that were given CPA + anti-CD8a antibody showed complete depletion of circulating CD8 T-cells after 2 antibody doses. The depletion was maintained for many weeks after antibody treatment (blue arrow heads below x-axis) was halted. CD8 T-cells levels were not detected in the CPA + anti-CD8a group at any of the 8 time points analyzed (small triangles superimposed on the x-axis, at time points from day 1 through day 50). Data shown are group mean values +/- SEM for n = 4 mice in each group.

Figure S9: FACS analysis during metro-CPA + Anti-IFNAR1 Treatment

A. Tumor-infiltrating CD8 T-cells

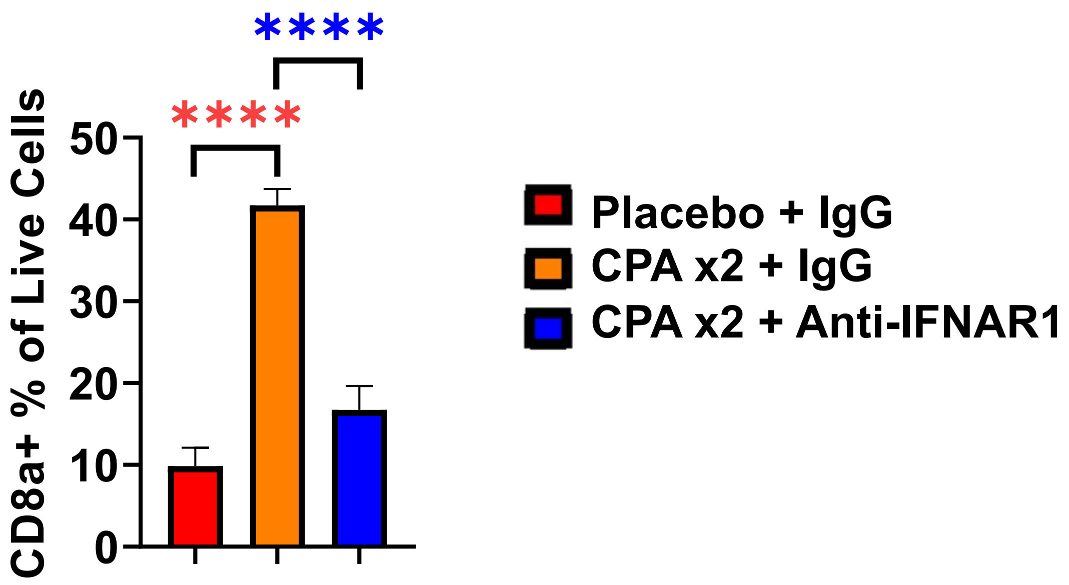

B. Circulating CD8 T-cells

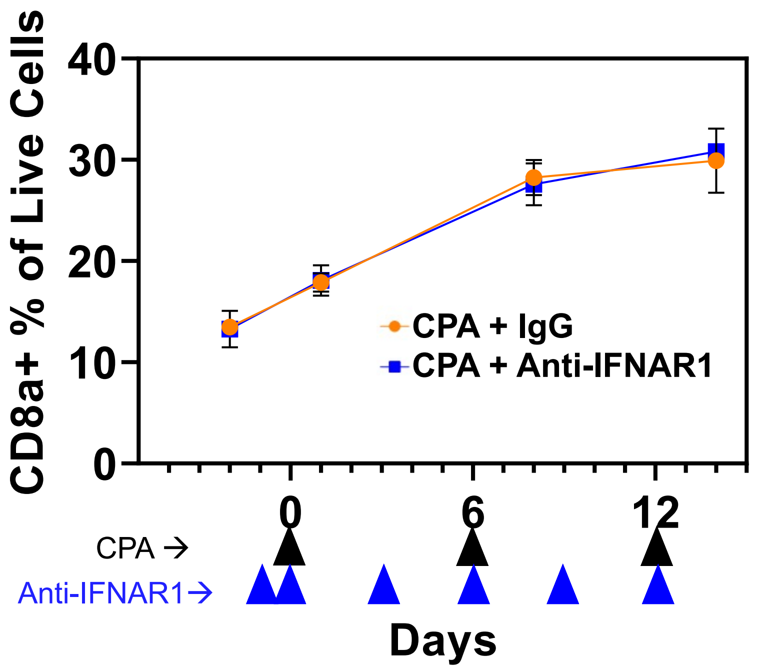

Fig. S9. FACS analysis of blood and tumors from mice given metronomic CPA treatment with and without anti-IFNAR1 antibody. **A**. FACS analysis on treatment day 12 of tumor infiltrating CD8 T-cells from the mice shown in Fig. 8. Anti-IFNAR1 antibody treatment almost completely blocked CD8 T-cells from infiltrating the tumors. Data shown are mean +/- SEM values for n = 4 for placebo + IgG, n = 5 for CPA + IgG, and n = 6 for CPA + Anti-IFNAR1. **B**. FACS analysis of circulating CD8 T-cells from Fig. 8 mice. Circulating CD8 T-cells were not significantly different in the CPA + Anti-IFNAR1 group versus the CPA + IgG group. Data based on n = 3 for each group.
